# A Robust Metal‐Organic Framework With ‘Molecular Gates’ for Efficient Separation of Ethane From Ethylene

**DOI:** 10.1002/anie.6088623

**Published:** 2026-04-15

**Authors:** Shao‐Min Wang, Liping Zhang, Yitao Li, Ziluo Fang, Xue Han, Stephen P. Thompson, Qing‐Yuan Yang, Sihai Yang

**Affiliations:** ^1^ State Key Laboratory of Fluorine & Nitrogen Chemicals School of Chemical Engineering and Technology Xi'an Jiaotong University Xi'an Shaanxi China; ^2^ Department of Chemistry University of Manchester Manchester UK; ^3^ MOE Key Laboratory of Bioinorganic and Synthetic Chemistry School of Chemistry Sun Yat‐Sen University Guangzhou China; ^4^ College of Chemistry Beijing Normal University Beijing China; ^5^ Diamond Light Source Harwell Science and Innovation Campus Didcot UK; ^6^ College of Chemistry and Molecular Engineering Beijing National Laboratory For Molecular Sciences Peking University Beijing China

**Keywords:** ethane/ethylene separation, gas adsorption, host–guest interactions, metal‐organic frameworks, molecular gates

## Abstract

Ethane‐selective sorbents can enable single‐step purification of ethylene but remain elusive to date. We report a Zn‐based metal‐organic framework (**ZAI‐3N**) decorated with amino‐based ‘molecular gates’ that exhibit highly selective adsorption of C_2_H_6_ over C_2_H_4_. Upon activation, **ZAI‐3N** (zinc‐adenine‐3‐aminoisonicotinic acid) features contractions of both Zn─N bonds and pores (from 2.009 to 1.914 Å and from 4.88 × 3.40 to 3.47 × 2.75 Å^2^, respectively). At 313 K and 1 bar, **ZAI‐3N** exhibits an exceptional ratio of 10.6 for C_2_H_6_/C_2_H_4_ uptakes and a benchmark selectivity of 11.7, outperforming state‐of‐the‐art porous solids. Synchrotron X‐ray powder diffraction and modelling reveal that the methyl group in C_2_H_6_ can trigger amino rotation and facilitate gate opening, while π‐electrons of C_2_H_4_ hinder such a process with a notably increased barrier (～5 and 11 kJ mol^−1^, respectively). Dynamic breakthrough experiments confirm the efficient separation of C_2_H_6_/C_2_H_4_ (v/v = 5/5 and 1/9), affording C_2_H_4_ with a high purity of 99.4% in single step with excellent recyclability and an C_2_H_4_ productivity of 10.3 mL g^−1^. This work demonstrates the judicious choice of ‘molecular gate’ as a promising protocol for challenging industrial gas separations.

## Graphical abstract

By installing an amino‐based molecular gate at a specific site within a robust zinc‐adenine framework, temperature‐tunable inversion of C_2_H_6_/C_2_H_4_ selectivity is achieved, enabling single‐step on‐stream production of polymer‐grade ethylene.

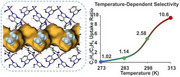

## 1 Introduction

Over 210 million tonnes of polymer‐grade ethylene (C_2_H_4_) are required annually. State‐of‐the‐art separation of ethylene/ethane (C_2_H_4_/C_2_H_6_) relies on cryogenic distillation, accounting for 0.3% of global energy consumption [1, 2, 3]. Adsorption‐based separation is a promising alternative with a much‐reduced carbon footprint, but the search for suitable sorbents remains elusive [4, 5, 6, 7, 8, 9, 10]. Metal‐organic frameworks (MOFs) exhibit tuneable pore environment and high surface areas (up to ∼7500 m^2^ g^−1^) [11, 12, 13, 14, 15, 16, 17]. However, most MOFs preferentially adsorb C_2_H_4_ over C_2_H_6_ due to the larger quadrupole moment and π‐electrons of C_2_H_4_, leading to complex purification processes of C_2_H_4_ [2, 18]. To achieve direct purification of C_2_H_4_, C_2_H_6_‐selective sorbents are highly desirable, particularly with high concentration of C_2_H_4_ [19, 20]. State‐of‐the‐art sorbents include Fe_2_(O_2_)(dobdc) (dobdc = 2,5‐dioxido‐1,4‐benzenedicarboxylate) [21], Cu(Qc)_2_ [22], and MAF‐49 [23], which show inverse C_2_H_6_/C_2_H_4_ selectivities of 4.4, 3.4, and 2.7, respectively. Flexible MOFs show external stimuli‐induced structural changes, which can afford enhanced selectivity through adaptive host–guest interactions [14, 24, 25, 26, 27, 28, 29]. To date, only a limited number of flexible MOFs have demonstrated ethane‐selective behavior for C_2_H_6_/C_2_H_4_ separation [24, 30, 31]. For instance, ZIF‐7 [24] shows C_2_H_6_/C_2_H_4_ uptake ratio of only ∼1.02 at 298 K, while X‐dia‐1‐Ni_0.89_Co_0.11_ [30], despite exhibiting an uptake ratio of 9.11, operates at 273 K. The utilization of ‘molecular gate’ in robust MOFs to exploit subtle differences in C_2_H_6_ and C_2_H_4_ is a promising approach to dictate selectivity driven by variation of activation energies.

**SCHEME 1:**
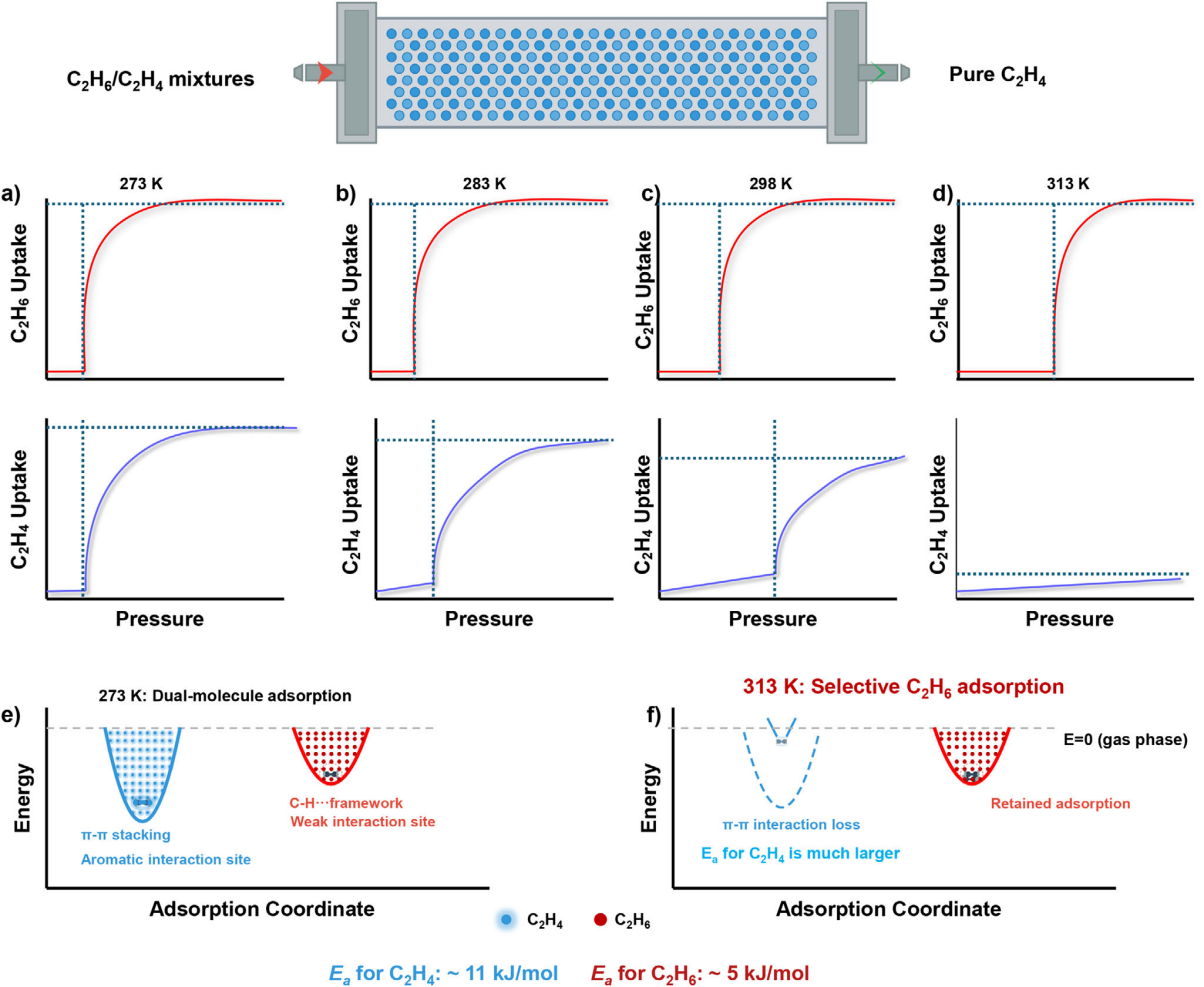
Thermally tuneable preferential binding of C_2_H_6_ over C_2_H_4_ in **ZAI‐3N**. (a–d) Adsorption isotherms showing temperature‐induced selectivity inversion from 273 to 313 K. (e, f) Schematic energy diagrams illustrating the mechanism: dual‐molecule adsorption at 273 K via π‐π stacking versus selective C_2_H_6_ adsorption at 313 K through disrupted π‐π stacking and retained C‐H⋯π interactions.

Herein, we report a systematic investigation of a series of Zn‐based MOFs, denoted as **ZAI** (Zinc–Adenine–Isonicotinic acid), **ZAI‐2N** (2‐aminoisonicotinic acid), **ZAI‐3M** (3‐methylisonicotinic acid), and **ZAI‐3N** (3‐aminoisonicotinic acid) for inverse C_2_H_6_/C_2_H_4_ separation. We demonstrate that the introduction of 3‐amino group (**ZAI‐3N**) at the target site as ‘molecular gate’ within zinc‐adenine MOFs can achieve an exceptional selectivity of 11.7 for C_2_H_6_/C_2_H_4_. The results reveal that 3‐amino functionalization uniquely enables temperature‐dependent gating through modulation of host‐guest interactions. At 273 K, co‐adsorption of C_2_H_6_/C_2_H_4_ occurs, promoted by π⋯π stacking and weak C─H⋯π interactions with the C_2_H_6_/C_2_H_4_ uptake ratio of only 1.02. By contrast, at 313 K, C_2_H_4_ adsorption is restricted due to hindered π⋯π stacking interactions, while retained C─H⋯π interactions promote selective binding of C_2_H_6_, achieving an exceptional C_2_H_6_/C_2_H_4_ uptake ratio of 10.6 (Scheme 1), outperforming state‐of‐the‐art porous solids. Dynamic breakthrough experiments enable single‐step production of polymer‐grade C_2_H_4_ (purity >99.4%) with excellent recyclability.

## 2 Results and Discussion

**FIGURE 1:**
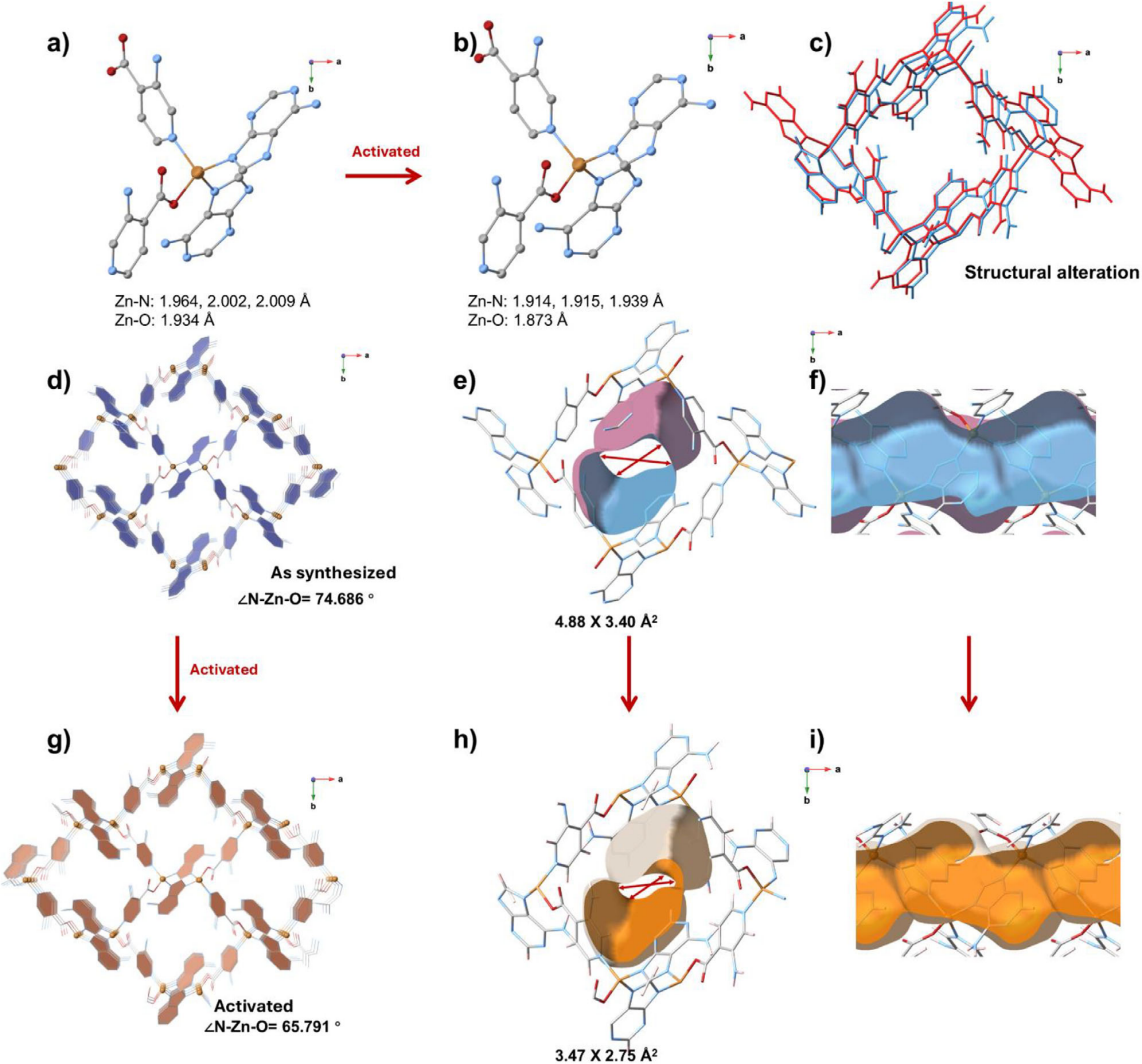
Structural characterization of **ZAI‐3N** activation. (a, b) Coordination geometry before and after activation. (c) Structural overlay showing framework alteration. (d, g) Crystal structures along c‐axis. e,h) Pore dimensions change from 4.88 × 3.40 Å^2^ (as‐synthesized) to 3.47 × 2.75 Å^2^ (activated). f, i) Channel cross‐sections showing gate‐narrowing effect. Blue represents as‐synthesized state; orange represents activated state.

Four isostructural MOFs (**ZAI**, **ZAI‐2N**, **ZAI‐3M**, and **ZAI‐3N**) were synthesized via solvothermal reactions of adenine with Zn(NO_3_)_2_·6H_2_O and corresponding isonicotinic acid derivatives in DMF. Single‐crystal X‐ray diffraction reveals that all four MOFs crystallize in orthorhombic space group *P*na2_1_ with a rare ***dmp*** topology featuring one‐dimensional channels (Figures 1a–c, S1–S3). Each Zn(II) center adopts a distorted tetrahedral coordination geometry, coordinated by one pyridyl nitrogen and one carboxylate oxygen from two distinct isonicotinate linkers, together with two nitrogen atoms from two adenine ligands (Zn–N/O: 1.934–2.015 Å; Figure 1d). The substituent position on isonicotinate modulates the pore environment. In 3‐substituted derivatives (**ZAI‐3M** and **ZAI‐3N**), functional groups project freely into the channel interior, enabling hydrogen bonding with guests. In **ZAI‐2N**, the 2‐amino group lies adjacent to the carboxylate, forming intramolecular hydrogen bonds that reduce guest accessibility. These distinctions render **ZAI‐3N** most favorable for C_2_H_6_ binding. Phase purity has been confirmed by powder X‐ray diffraction (PXRD; Figures S4–S9). Variable‐temperature PXRD confirms that the structural integrity of **ZAI‐3N** is retained up to 300°C (Figure S9). Thermogravimetric analysis (TGA) shows that all materials undergo initial weight loss above 220°C (Figures S12–S15), which is attributed to the removal of residual guest molecules trapped in the pore. Rietveld refinements of activated **ZAI‐3N‐a** (Figures S10, S11) reveal coordination contraction with Zn─N/O bonds shortening from 1.934–2.002 to 1.873–1.915 Å and ∠N‐Zn‐O decreasing from 74.69° to 65.79° (Figure 1e). This narrows the pore aperture from 4.88 × 3.40 to 3.47 × 2.75 Å^2^, enabling temperature‐responsive C_2_H_6_/C_2_H_4_ separation. Gas adsorption measurements confirm the permanent porosity of all four materials. **ZAI** exhibits a type‐I N_2_ isotherm at 77 K with a Brunauer–Emmett–Teller (BET) surface area of 818 m^2^ g^−1^ (Figures S16, S17). In contrast, **ZAI‐3M**, **ZAI‐2N**, and **ZAI‐3N** show little N_2_ uptake at 77 K but display type‐I microporous behavior in CO_2_ adsorption isotherms at 195 K, yielding BET surface areas of 638, 451, and 486 m^2^ g^−1^, respectively (Figures S18–S26). The negligible N_2_ adsorption at 77 K for these three materials is attributed to framework flexibility and kinetic restrictions at cryogenic temperatures.

**FIGURE 2:**
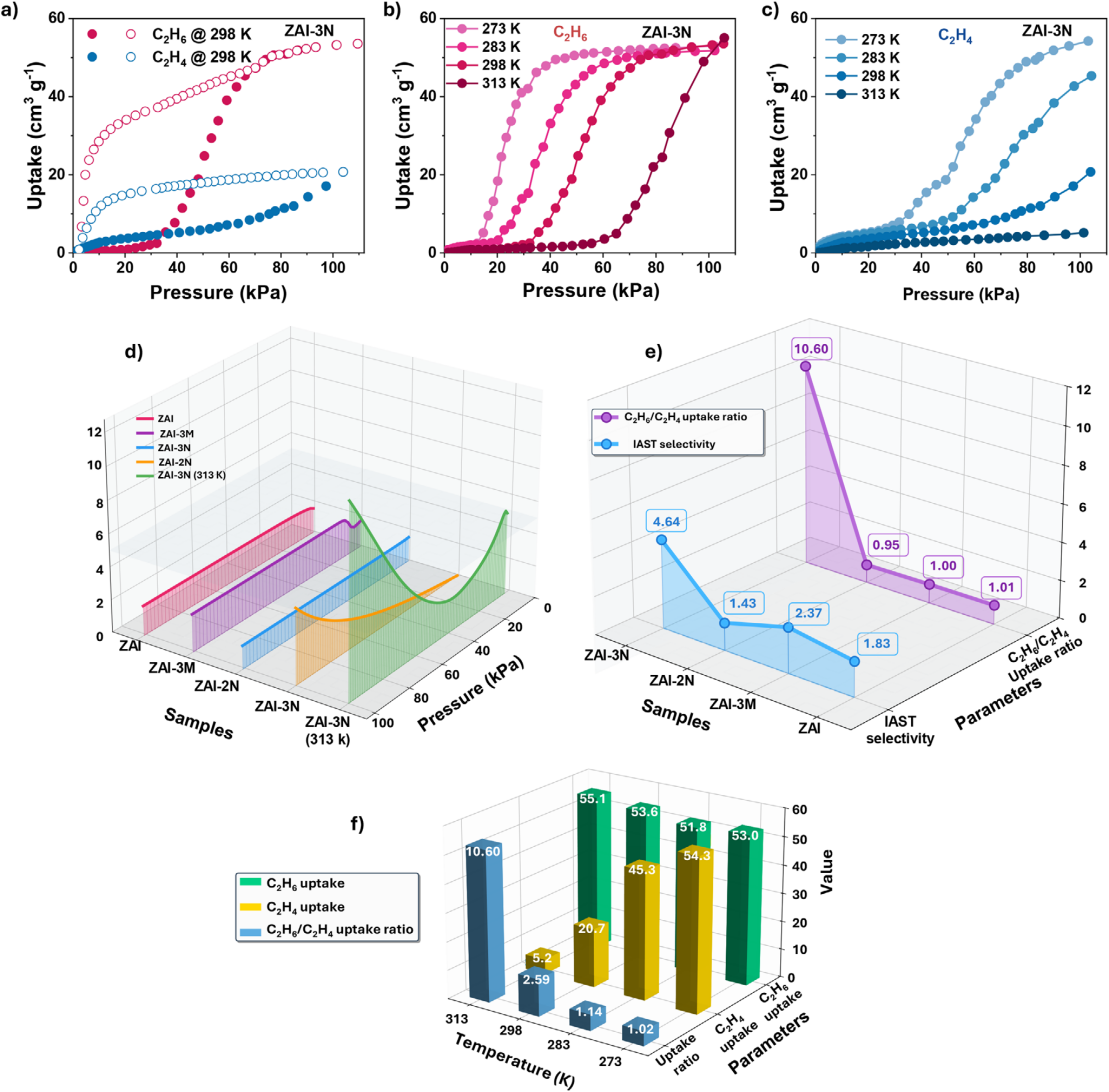
Gas adsorption and separation performance of ZAI materials. (a–c) C_2_H_6_ and C_2_H_4_ sorption isotherms of **ZAI‐3N** recorded between 273 and 313 K. (d) 3D visualization of adsorption isotherms across different **ZAI** samples and temperatures. (e) Comparison of C_2_H_6_/C_2_H_4_ uptake ratio and IAST selectivity, showing **ZAI‐3N**’s superior performance (uptake ratio: 10.6). (f) Influence of temperature on individual gas capacities and overall selectivity of **ZAI‐3N**.

Pure‐gas adsorption measurements of C_2_H_4_ and C_2_H_6_ uncovered markedly different sorption characteristics across the ZAI series. **ZAI‐3N‐a** exhibits an intriguing temperature‐responsive adsorption behavior. At 298 K, **ZAI‐3N** preferentially takes up C_2_H_6_ (53.6 cm^3^ g^−1^) following a gate‐opening near 33 kPa; in contrast, C_2_H_4_ uptakes remain modest (20.7 cm^3^ g^−1^), giving a capacity ratio of 2.6 (Figure 2a). Adsorption data collected over 273–313 K reveal a pronounced, progressive increase in C_2_H_6_ preference (Figure 2b,c). Notably, C_2_H_6_ triggers the gate‐opening transition at every temperature studied, achieving a capacity of 55.1 cm^3^ g^−1^ (313 K, 1 bar), whereas C_2_H_4_ adsorption drops to 5.2 cm^3^ g^−1^ under the same condition. The resulting C_2_H_6_/C_2_H_4_ uptake ratio reaches a remarkable value of 10.6 at 313 K. Unlike ZIF‐7 [24], which shows similar gate‐opening pressures for both gases, **ZAI‐3N** achieves selective gate‐opening to C_2_H_6_, thereby binding it selectively. Five consecutive adsorption–desorption runs verify complete regenerability without loss of capacity (Figure S27).

**FIGURE 3:**
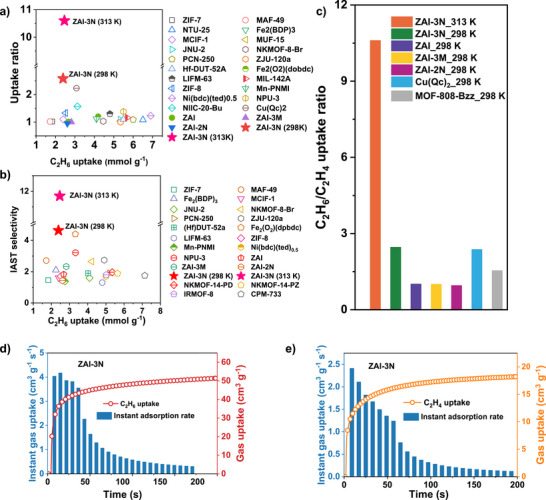
**ZAI‐3N** benchmarking against ethane‐selective sorbents in literature: (a) IAST selectivity plotted against C_2_H_6_ capacity and (b) C_2_H_6_/C_2_H_4_ uptake ratio as a function of C_2_H_6_ loading, (c) Bar‐chart comparison of uptake ratios for all **ZAI** variants and leading reference materials at 100 kPa. (d, e) Time‐resolved kinetic profiles illustrating the faster sorption rate of C_2_H_6_ relative to C_2_H_4_.

By contrast, the parent **ZAI** framework adsorbs both gases in essentially equal amounts (C_2_H_6_/C_2_H_4_ ratio of 1.01, 298 K, Figure S28). **ZAI‐3M** shows a marginal C_2_H_6_ preference in the low‐pressure regime that vanishes upon saturation (Figure S29), and **ZAI‐2N** displays a mild C_2_H_4_ preference (Figure S30). This comparison demonstrates that the 3‐position of amino group is critical to promoting selective adsorption of C_2_H_6_, as it projects into the pore and serves as a molecular gate that preferentially responds to the more polarizable C_2_H_6_, while the 2‐amino group is sterically constrained near the metal center. Ideal adsorbed solution theory (IAST) calculations reveal an exceptional selectivity of 11.7 for **ZAI‐3N** at 313 K (Figure 2d), representing a benchmark for porous materials reported to date. Even under an industrial feed ratio of C_2_H_6_/C_2_H_4_ (1:9), **ZAI‐3N** exhibits a superior selectivity of 4.41, whereas leading materials show selectivities of 1.44–2.34 (Figures S31–S34), demonstrating the practical potential of **ZAI‐3N**. Compared with reported ethane‐selective MOFs, **ZAI‐3N** (313 K) exhibits both high C_2_H_6_ uptake and exceptional selectivity, positioning it in the upper‐right region of the performance map (Figure 3a,b). The uptake ratio of 10.6 at 313 K surpasses all leading materials, including ZIF‐7, Cu(Qc)_2_ (Figure 3c). Kinetic analysis at 298 K reveals favorable adsorption with preferential C_2_H_6_ rates (3.56‐4.05 cm^3^ g^−1^ s^−1^) versus C_2_H_4_ (1.67‐2.41 cm^3^ g^−1^ s^−1^) (Figure 3e,f), indicating synergistic kinetic and thermodynamic selectivity for their dynamic separation.

**FIGURE 4:**
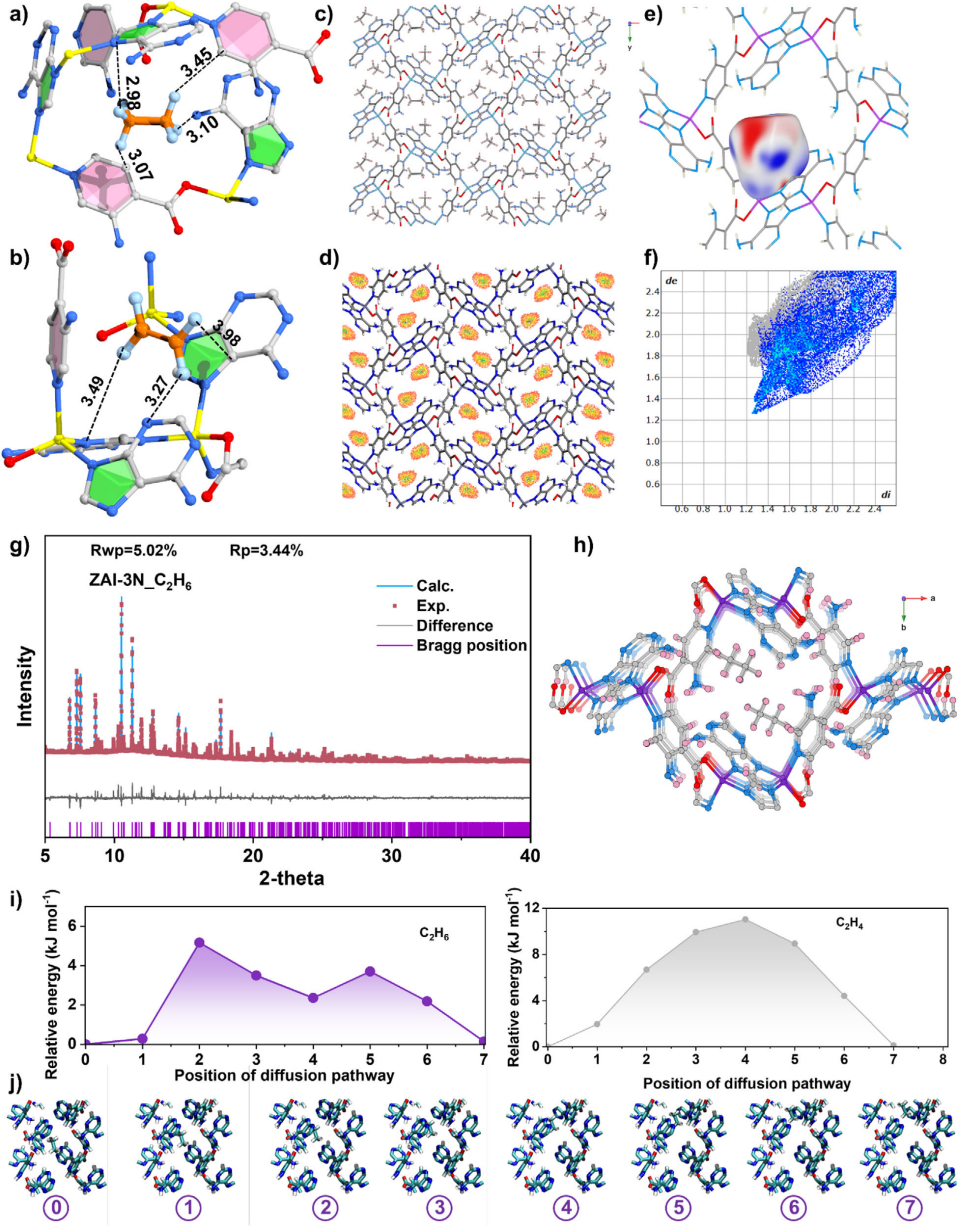
Host–guest interactions governing C_2_H_6_/C_2_H_4_ discrimination in **ZAI‐3N**. Optimized binding geometries with characteristic interatomic distances (Å) for (a) C_2_H_6_ and (b) C_2_H_4_. GCMC‐derived probability maps for adsorbed C_2_H_6_ in **ZAI‐3N**: (c) spatial occupancy and (d) number‐density contour. (e) Hirshfeld surface mapped onto the adsorbed C_2_H_6_ molecule. (f) Two‐dimensional fingerprint plot highlighting the principal contact types. (g) Observed, calculated, and difference profiles from Rietveld fitting of synchrotron PXRD data [λ = 0.824495(1) Å] for C_2_H_6_‐loaded **ZAI‐3N**. (h) Crystallographic positions of adsorbed C_2_H_6_ molecules in **ZAI‐3N** derived from SPXRD refinements. (i) Calculated energy landscape along the C_2_H_6_ and C_2_H_4_ migration coordinate, (j) snapshots depicting amino‐group rotation that enables C─H⋯NH_2_ contacts during C_2_H_6_ diffusion.

Computational and structural analyses based on Grand Canonical Monte Carlo (GCMC) simulations reveal the molecular details of selective binding of C_2_H_6_ in **ZAI‐3N** (Figure 4). Adsorbed C_2_H_6_ molecules form multiple C─H⋯NH_2_ contacts at 2.98–3.45 Å while C_2_H_4_ exhibits weaker interactions with a longer binding distance (3.27–3.98 Å) (Figure 4a,b). **ZAI**, **ZAI‐3M**, and **ZAI‐2N** show similar binding distances (2.87–3.17 Å) for both molecules (Figure S35), as **ZAI** lacks functional groups, **ZAI‐3M** bears non‐hydrogen‐bonding methyl groups, and **ZAI‐2N** has 2‐positioned amino groups that are sterically hindered with reduced accessibility, confirming the unique role of 3‐positioned amino groups. C_2_H_6_ molecules primarily interact with aromatic rings (3.00–3.43 Å) rather than amino groups directly (Figure S35). This limited ─NH_2_ accessibility accounts for the lower C_2_H_6_/C_2_H_4_ selectivity compared with the 3‐amino analogue. Moreover, GCMC simulations reveal preferential ethane binding near amino groups (Figure 4c,d), with Hirshfeld surface analysis identifying dominant interaction types, where H⋯H contacts contribute the largest proportion, followed by H⋯N hydrogen bonding interactions (Figure 4e,f). Rietveld analysis of synchrotron X‐ray powder diffraction of the C_2_H_6_‐loaded material converges to *R*_wp_ = 5.02%, confirming the accuracy of the host‐guest structural model and pinpointing ethane at the amino binding pockets (Figure 4g,h). Energy barrier calculations reveal key insights (Figure 4i): ethane encounters a moderate barrier (∼5 kJ/mol) at amino positions where rotational freedom enables C─H⋯NH_2_ interactions (Figure 4j), while ethylene faces a much higher barrier (∼11 kJ/mol, Figure S36) owing to the lack of notable interactions. Elevated temperatures widen this energetic gap, giving rise to thermally activated molecular gating driven by amino‐group libration.

**FIGURE 5:**
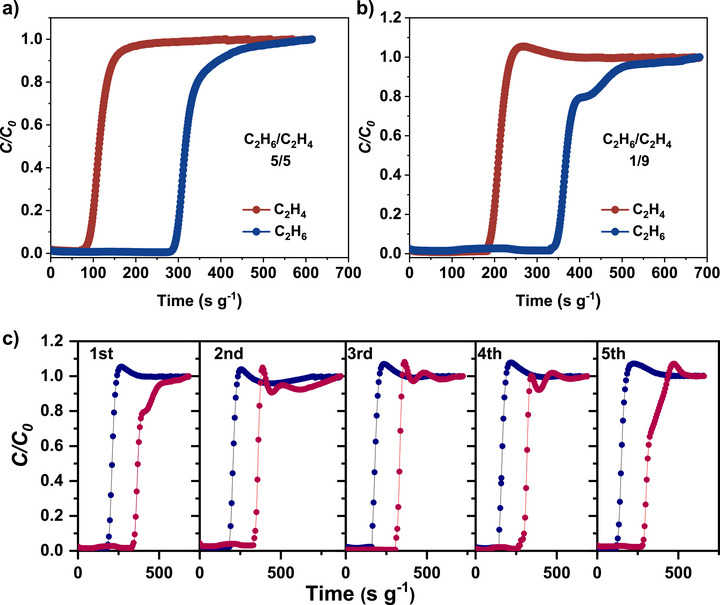
Fixed‐bed breakthrough behavior of **ZAI‐3**N. Breakthrough curves for C_2_H_6_/C_2_H_4_ binary mixtures with ratios of (a) 5:5 (6 mL min^−1^) and (b) 1:9 (8 mL min^−1^) at 298 K and 1 bar. (c) Overlay of individual‐cycle breakthrough curves confirming reproducible C_2_H_6_/C_2_H_4_ fractionation across successive runs.

Dynamic breakthrough experiments at 298 K, a temperature more relevant to practical applications and comparable with literature benchmarks, reveal efficient gas discrimination under flow conditions for **ZAI‐3N** (Figure 5). Under a 5/5 (v/v) feed of C_2_H_6_/C_2_H_4_, C_2_H_4_ elutes first at approximately 80.5 s g^−1^, whereas C_2_H_6_ is retained until 286 s g^−1^, delivering a high‐purity C_2_H_4_ effluent (Figure 5a). The dynamic selectivity of C_2_H_6_ over C_2_H_4_ was calculated to be 2.7, with a C_2_H_4_ productivity of 10.3 L kg^−1^, which is comparable to the benchmark MOFs reported to date, such as MUF‐15 (14.0 L kg^−1^) [32], Cu(Qc)_2_ (4.3 L kg^−1^) [22]. Importantly, this selective behavior retains under industrially relevant feed compositions (C_2_H_6_/C_2_H_4_ = 1:9), where the framework maintains ethane retention despite dilute concentrations (Figure 5b). Repeated cycling over five successive runs confirms stable column performance, with nearly superimposable breakthrough profiles attesting to long‐term operational reliability (Figure 5c). Furthermore, breakthrough experiments at 313 K confirm that **ZAI‐3N** retains clear separation, albeit with a reduced difference in retention time due to the higher gate‐opening pressure at 313 K (Figure S38). Water vapor adsorption reveals minimal uptake at low relative pressures (∼5.7 cm^3^ g^−1^ at *P*/*P*_0_ = 0.05, Figure S39), suggesting good tolerance to low‐humidity conditions. The dynamic study demonstrates the promising potential of molecular gating effect of **ZAI‐3N** in single‐step separation of C_2_H_6_/C_2_H_4_.

## 3 Conclusion

We demonstrate that the judicious positioning of an amino group in robust MOFs can enable highly selective adsorption of C_2_H_6_ over C_2_H_4_ through the molecular gating effect. **ZAI‐3N** exhibits an exceptional IAST selectivity of 11.7 for C_2_H_6_/C_2_H_4_, exceeding all porous sorbents to date. The separation relies on different activation barriers: C─H⋯NH_2_ interactions in ethane (∼5 kJ/mol) trigger amino rotation and gate opening, while ethylene's π‐electrons encounter prohibitive barriers (∼11 kJ/mol). Breakthrough experiments confirm practical performance, producing polymer‐grade ethylene with a purity of >99.4% with excellent stability. Strategic functionalization with ‘molecular gate’ can uncover unusual adsorption selectivity, highly desired for tackling challenging industrial gas separations. This ‘molecular gating’ strategy could be extended to other industrially challenging separations, such as C_3_H_8_/C_3_H_6_, C_2_H_2_/C_2_H_4_, and natural gas upgrading, by tailoring the nature and position of functional groups and rotational barriers at the pore aperture, offering new design principles for next‐generation sorbent materials.

## Conflicts of Interest

The authors declare no conflicts of interest.

## Supporting information




**Supporting File 1**: anie72217‐sup‐0001‐SuppMat.docx.

**Supporting File 2**: anie72217‐sup‐0002‐Data.zip.

## Data Availability

The data that support the findings of this study are available in the Supporting Information of this article and/or available from the corresponding authors upon reasonable requests.
