# Supplementary material for: A Robust Metal‐Organic Framework With ‘Molecular Gates’ for Efficient Separation of Ethane From Ethylene
Source: Angew Chem Int Ed Engl. 2026 Apr 15;65(21):e6088623. doi: 10.1002/anie.6088623 (PMC13182193; doi:10.1002/anie.6088623)
Supplement: Supplementary file 1 — Supporting File 1: anie72217‐sup‐0001‐SuppMat.docx. [file ANIE-65-e6088623-s001.docx]

Supplementary Information for

A Robust Metal-Organic Framework with ‘Molecular Gates’ for Efficient Separation of Ethane from Ethylene

Shao-Min Wang, ^[a, b]^ Liping Zhang, ^[a]^  Yitao Li, ^[a]^ Ziluo Fang, ^[c]^ Xue Han, ^[d]^ Stephen P. Thompson, ^[e]^ Qing-Yuan Yang*^[a]^ and Sihai Yang*^[b,f]^

[a] State Key Laboratory of Fluorine & Nitrogen Chemicals, School of Chemical Engineering and Technology, Xi’an Jiaotong University, Xi’an, Shaanxi 710049, China

[b] Department of Chemistry, University of Manchester, Manchester, M13 9PL, UK

[c] MOE Key Laboratory of Bioinorganic and Synthetic Chemistry, School of Chemistry, Sun Yat-Sen University, Guangzhou, 510275 China

[d] College of Chemistry, Beijing Normal University, 463 Beijing 100091, China

[e] Diamond Light Source, Harwell Science and Innovation Campus, Didcot, UK

[f] College of Chemistry and Molecular Engineering, Beijing National Laboratory for Molecular Sciences, Peking University, Beijing, 100871 China

E-mail: [Sihai.Yang@pku.edu.cn](mailto:Sihai.Yang@pku.edu.cn); qingyuan.yang@xjtu.edu.cn

Supplementary Text

**Materials and Methods**

Zinc nitrate hexahydrate (AR, ≥99.0%), isonicotinic acid (AR, 99%), Adenine (98%), 3-aminoisonicotinic Acid (98%), were obtained from Aladdin (Shanghai Aladdin Biochemical Technology Co., Ltd.). 3-Methyl-4-pyridinecarboxylic acid (97%) and 2-Aminoisonicotinic acid (97%) were purchased from Shanghai Bide Pharmatech Co., Ltd., N,N-Dimethylformamide (AR, 99.5%) was purchased from Shanghai Macklin Biochemical Co., Ltd. Dichloromethane (DCM, AR，≥99.5%) was purchased from Sinopharm Chemical Reagent Co., Ltd. UAll of the chemical reagents were commercially available on the market and were used without further purification.

Synthesis of **ZAI**

A remotely modified method for preparing **ZAI** was developed based on available literature.^[1]^ Typically, Zn(NO_3_)_2_·6H_2_O (0.30 g, 1 mmol), Adenine (0.14 g, 1 mmol), isonicotinic acid (0.123 g, 1 mmol) were dissolved in 6 mL of DMF in a 20 mL vial. After being sealed and ultrasonicated for 5 min, the consequent solution was transferred to a Teflon-lined autoclave and placed into the oven (120 °C) for 72 h, and natural cooling brought the temperature to room temperature. The resulting purple crystals were collected by filtration and washed with DMF. The samples of **ZAI** were solvent exchanged by DCM for 72 h and a vacuum oven was used to dry the product for 24 hours at 45°C. Yield: ∼80%.

Synthesis of **ZAI-3M**, **ZAI-2N**, and **ZAI-3N**

These three MOFs were synthesized using the same solvothermal method as ZAI. Zn(NO_3_)_2_·6H_2_O (0.30 g, 1 mmol), adenine (0.14 g, 1 mmol), and the corresponding isonicotinic acid (1 mmol) were dissolved in 6 mL DMF and heated at 120 °C for 72 h. The isonicotinic acid derivatives used were 3-methyl-4-pyridinecarboxylic acid for ZAI-3M, 2-aminoisonicotinic acid for ZAI-2N, and 3-aminoisonicotinic acid for ZAI-3N. Yields: ~63% (ZAI-3M), ~56% (ZAI-2N), and ~71% (ZAI-3N), based on Zn.

**Physical Measurements**

Powder X-ray diffraction (PXRD) data was recorded using a Bruker D8 Advance. A NETZSCH STA449F5 thermal analyzer (Germany) was used for thermogravimetric analysis (TGA). Thermo Scientific Nicolet iS50 was utilized for Fourier transform infrared (FT-IR) spectroscopy. The single crystal data were collected on a Bruker D8 Venture diffractometer with Cu/Mo-Kα radiation (λ = 1.54178, 0.71073 Å) diffractometer. Single crystal data collection was conducted on a Bruker D8 Venture single-crystal X-ray diffractometer at 100 K, using Mo Kα X-rays (λ = 0.71073 Å) generated at 50 kV and 1.4 mA. The structures were solved by SHELXS (direct methods) and refined by SHELXL (fullmatrix least-squares techniques) in the Olex2 package.^[2-3]^

**Gas Adsorption Measurements**

Low-pressure C_2_H_6_ and C_2_H_4_ sorption experiments were carried out on JW-BK200C (JWGB Sci & Tech Co. Ltd.) at different temperatures (273 to 298 K). Before gas adsorption measurements, the activated MOF samples were obtained by heating at 323 K under dynamic vacuum for 12 hours. The temperatures during sorption measurements were precisely maintained using anhydrous ethanol as a constant temperature bath. 77 K liquid nitrogen and 87 K liquid argon baths were used to maintain the cryogenic temperatures.

**Breakthrough experiments**

With dynamic breakthrough equipment (BSD-MAB, Beishide Instrument Technology, Beijing Co. Ltd.), dynamic breakthrough experiments were carried out at 100 kPa and 298 K. For this, C_2_H_6_/C_2_H_4_ gas mixtures (5/5, 1/9, v/v) were used. A glass column filled with activated MOF samples was used for the experiments. Before breakthrough experiments, the columns were degassed for 12 hours at 40 °C under vacuum. Using a gas flow meter, the total C_2_H_6_/C_2_H_4_ gas flow rate at the entrance was set to 6 mL min^-1^. Gases emitted from the adsorption bed were continuously monitored using a BSD mass spectrometer (thermal conductivity detector (TCD), 1 ppm detection limit).

**Rietveld refinement and structure determination**

High-resolution X-ray powder diffraction of ZAI-3N-a and ZAI-3N@C_2_H_6_ were carried out on beamline I11 of the Diamond Light Source [λ = 0.826844(2) Å], and TOPAS-5 was used for Pawley and Rietveld refinements.^[4]^ The data collected at 298 K were used for the extraction of binding sites via Rietveld refinement using Material Studio 2019. The structure model of the framework was established based on the single-crystal structure with restraints and constraints applied to the linker. The six-membered ring in the linker was described using a semi-rigid body with fixed bond angles for C–C–C, C–N–C, and C–C–C_carboxyl_ at 120°. The C–N and C–C bond distances in the six-membered ring were fixed at 1.390 Å. The remaining C–C, C–H, and C–O bond distances were allowed to be refined within a chemically sensible range. The atomic displacement parameters (ADPs) were defined on an elemental basis using isotropic parameters (Uiso). The C_2_H_4_ and C_2_H_6_ were modeled using rigid bodies: the C=C bond distance was fixed at 1.337 Å (obtained through geometry optimization using the Compass II force field in the Forcite module), and the C–C bond distance was fixed at 1.519 Å (also obtained through geometry optimization using the Compass II force field in the Forcite module). For the Rietveld refinement, the structural parameters of the framework and the non-structural parameters (from Pawley refinement) were first fixed. The initial position of the center of mass and the orientation of the gas molecules were obtained by simulated annealing (SA). At the final stage, all structural/non-structural parameters were released and refined.

**IAST selectivity calculation details**

The single-component adsorption isotherms of C_2_H_6_ and C_2_H_4_ on MOF samples obtained at 298K were fitted using the dual-site Langmuir-Freundlich model.

$$q=q_{A,sat}\frac{b_{A}p^{{}_{A}}}{1+b_{A}p^{{}_{A}}}+q_{B,sat}\frac{b_{B}p^{{}_{B}}}{1+b_{B}p^{{}_{B}}}$$

where q represents the adsorbed capacity per mass of adsorbent (mmol g^-1^), $q_{A,sat}$ and $q_{B,sat}$ are the saturation uptake capacities at site A and site B, respectively, $b_{A}$ and $b_{B}$ represent the constant at adsorption site A and site B, respectively, $P$ represents the total pressure of the gas at the equilibrium (kPa) and represents the Freundlich exponent.

Adsorption selectivity of C_2_H_6_/C_2_H_4_, mixed gases were predicted from single component adsorption isotherms using Ideal Adsorbed Solution Theory (IAST).

$$S_{ads}=\frac{{X_{A}}/{X_{B}}}{{Y_{A}}/{Y_{B}}}$$

Where S is the selectivity of component A relative to B. X_A_ and X_B_ are the molar fractions of components A and B in the adsorption phase, respectively. Y_A_ and Y_B_ are molar fractions of components A and B in the gas phase, respectively.

**Theoretical calculations**

All the geometry optimizations and diffusion process calculations based on density functional theory (DFT) were performed using the projector-augmented wave method and the Perdew–Burke–Ernzerhof (PBE) functional as implemented in Vienna ab initio Simulation Package (VASP).^[5-6]^ The approach of Grimme (DFT-D3) with Becke–Jonson damping was adopted for the van der Waals (vdW) interaction.^[7-9]^ The energy cutoff for the plane-wave basis set was 520 eV. Brillouin zone was sampled by a 2×3×2 k-point grid by using a 1×1×2 supercell. The energy and force criteria for convergence were set to 1 × 10⁻⁷ eV and 0.03 eV/Å, respectively. The climbing image nudged elastic band method (CI-NEB) was adopted to investigate the minimum energy path (MEP) associated with the C_2_H_6_ and C_2_H_4_ transport in ZAI-3N via a jump sequence by passing the pore-apertures.^[10-11]^

**BET surface area calculation**

To determine the specific surface areas of the samples, the obtained N_2_ or CO_2_ isotherms at 77 or 195 K were calculated by using the Brunauer–Emmett–Teller (BET) surface identification (BETSI) method.^[12]^

**Computational details**

Before the Grand canonical Monte Carlo (GCMC) simulations, the guest molecules C_2_H_6_ and C_2_H_4_ were geometrically optimized using Forcite code^[13]^ and the electrostatic potential (ESP) charges were applied to the guest molecules. The initial framework structures, **ZAI, ZAI-3M, ZAI-2N** and **ZAI-3N** were adopted from the single-crystal structure and further geometrically optimized by using the Forcite module with a Universal force field.^[14]^ *Q*eq fitted charges were applied to the framework. The optimized structures matched well with the experimentally determined crystal structures. The Metropolis method^[15]^ was applied to perform the GCMC simulations. The standard Universal force field described the guest-framework interactions. For each state point, the system was equilibrated for 1×10^5^ steps, and then the ultimate data were collected for another 1×10^6^ steps. The task simulated the beneficial adsorption sites with a single guest molecule. Finally, the Fixed loading task was applied to evaluate the adsorption enthalpy at 298 K. The charge density difference between the guest molecules and framework were derived from density functional theory (DFT) calculations taking the generalized gradient approximation (GGA) with a Perdew-Burke-Ernzerh (PBE) as the exchange-correlation functional with the CASTEP package ^[6]^. The guest molecules were isolated from the framework for the charge density difference calculation. The cutoff energy was 380 eV.

**Table S1.** Comparison of molecular sizes and physical properties of ethane and ethylene.

| Compounds | Ball and stick model | Dimensions | Kinetic diameter (Å) | Boiling point (K) | Polarizability  (Å3) | Quadrupole  moment×10^26^/esu cm^2^ |
| --- | --- | --- | --- | --- | --- | --- |
| Ethane  (C_2_H_6_) | 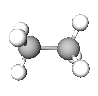 | 3.81×4.08×4.82 | 4.4 | 184.6 | 4.43-4.47 | +0.65 |
| Ethylene  (C_2_H_4_) | 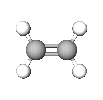 | 3.28×4.18×4.84 | 4.2 | 169.5 | 4.25 | +1.5 |


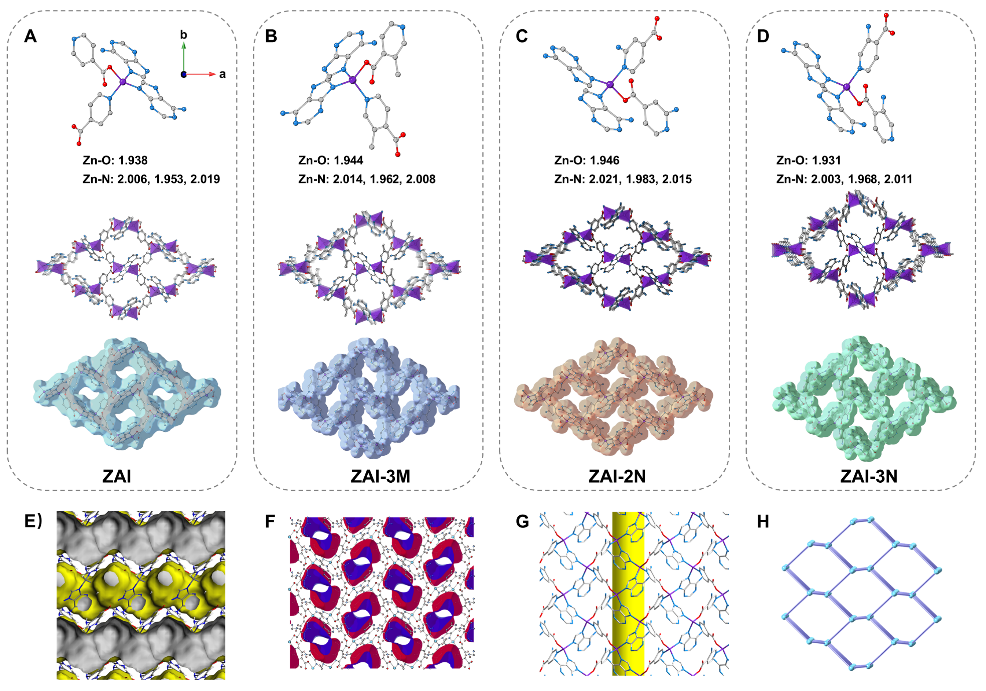


**Figure S1 Crystal structures and framework architectures of ZAI-based MOFs.** (A-D) Structural comparison of **ZAI**, **ZAI-3M**, **ZAI-2N**, and **ZAI-3N** showing coordination environments (top), 3D structures (middle), and van der Waals surface representations (bottom). Zn-O and Zn-N bond distances (Å) are indicated. Detailed structural analysis of **ZAI-3N**: (E) Connolly surface showing pore channels with accessible void space, (F) solvent-excluded surface, (G) 1D channel, and (H) dmp topological network.


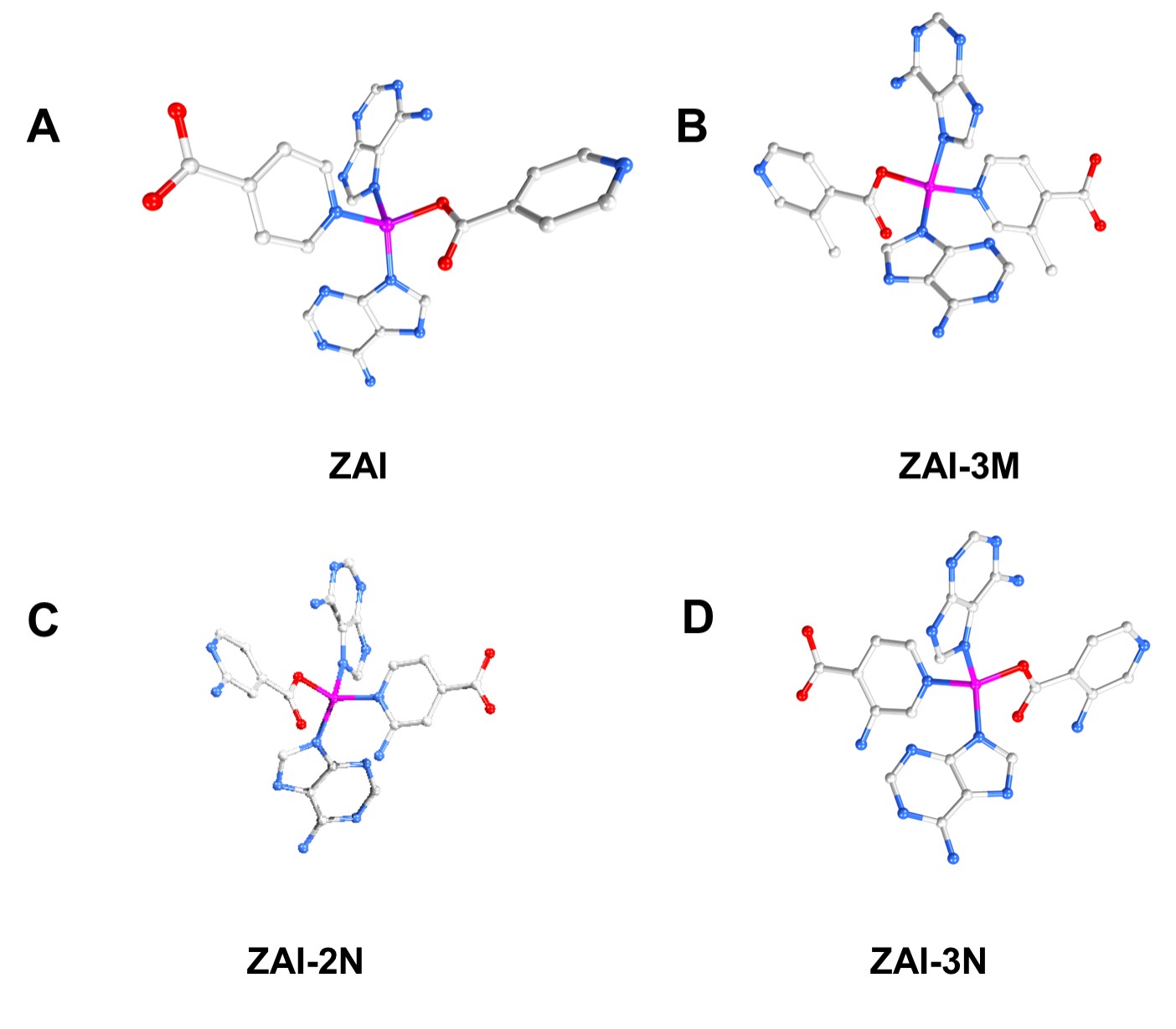


**Figure S2** The coordination environment of Zn(II) in (A) **ZAI**, (B) **ZAI-3M**, (C) **ZAI-2N**, (D) **ZAI-3N**.


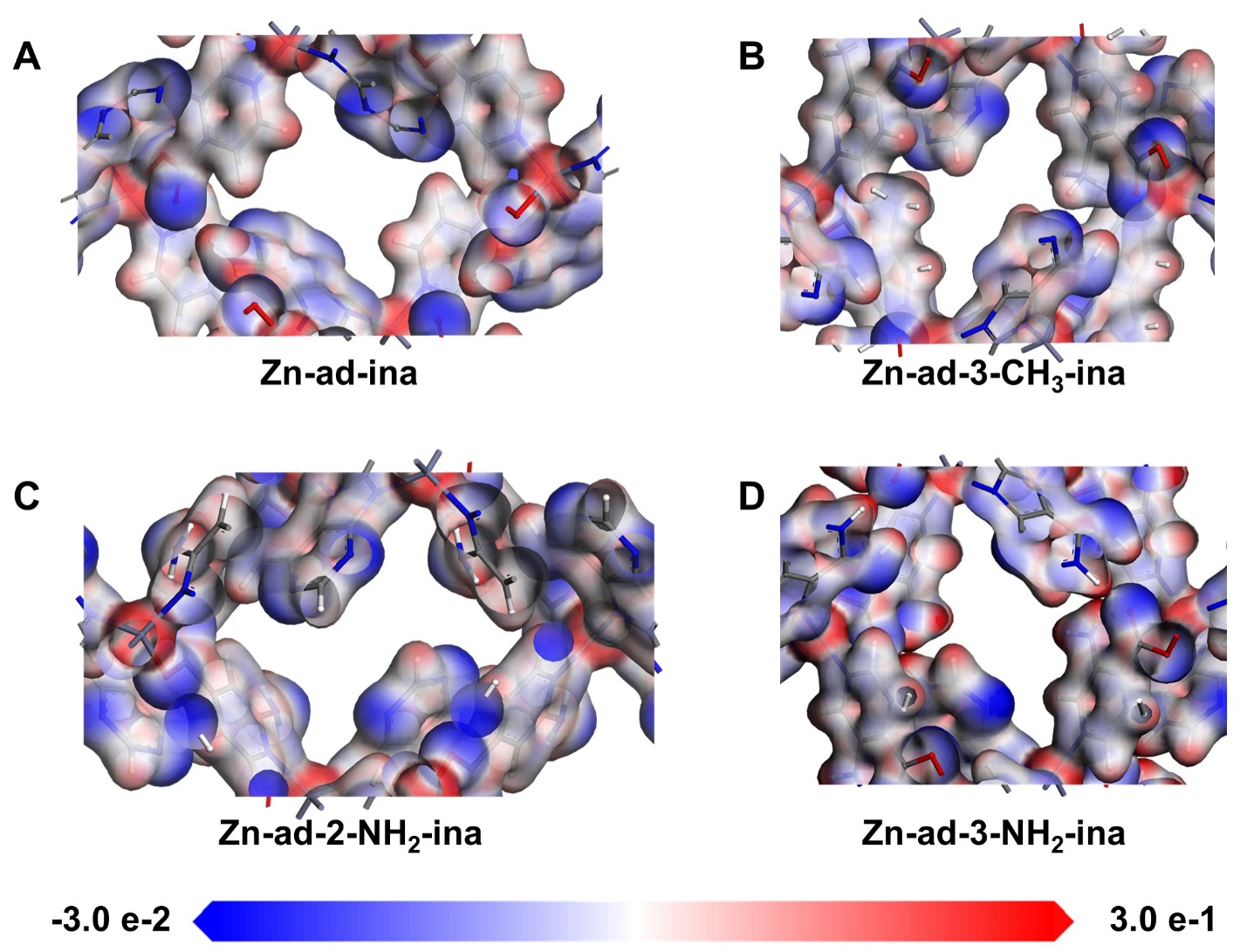


**Figure S3** The surface electrostatic potential of (A) **ZAI**, (B) **ZAI-3M**, (C) **ZAI-2N**, (D) **ZAI-3N**, mapped with a scale spanning from -0.03 Hartree/e (blue) through 0 to 0.3 Hartree/e (red).

**Figure S4** PXRD pattern of **ZAI**. Expected Bragg peaks are labelled with blue marks at the bottom.

**Figure S5** PXRD pattern of **ZAI-3M.** Expected Bragg peaks are labelled with blue marks at the bottom.

**Figure S6** PXRD pattern of **ZAI-2N.** Expected Bragg peaks are labelled with blue marks at the bottom.

**Figure S7** PXRD pattern of **ZAI-3N.**  Expected Bragg peaks are labelled with blue marks at the bottom.


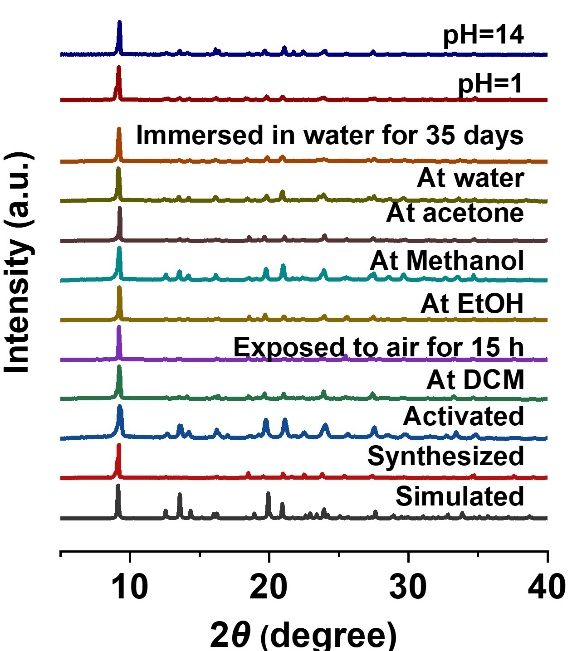


**Figure S8** PXRD patterns of **ZAI-3N**: simulated, as-synthesized, activated, and after exposure to various environmental conditions (DCM, air, ethanol, methanol, acetone, water) including extended water immersion (35 days), solution with pH=1, pH=14 (for 2 hours), demonstrating the high structural stability.

**Figure S9.** Variable-temperature PXRD patterns for ZAI-3N, demonstrating its structural stability (298-573 K).

**Figure S10** Rietveld refinement of desolvated **ZAI-3N**.


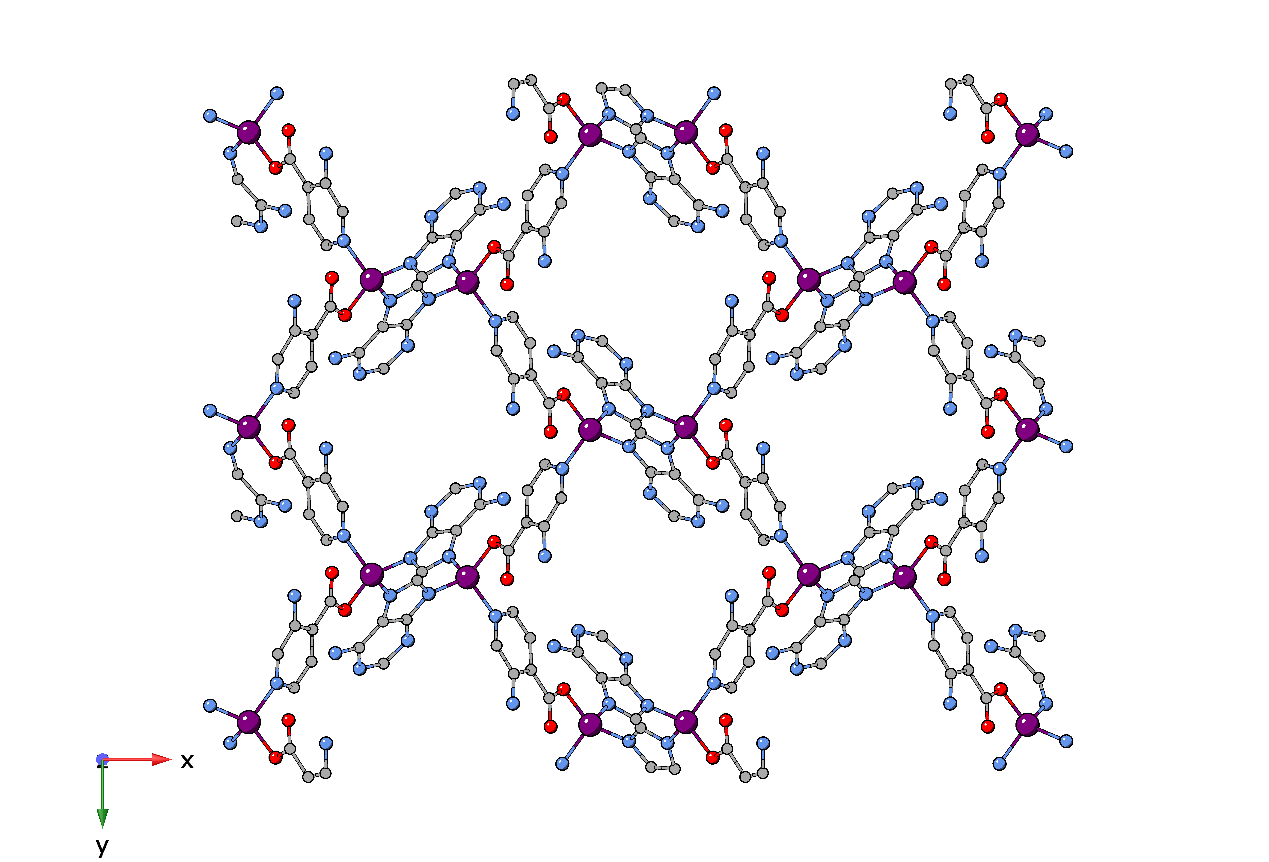


**Figure S11** Framework structure of desolvated **ZAI-3N** viewed along the c-axis

**Figure S12** TGA curve for **ZAI**.

**Figure S13** TGA curve for **ZAI-3M**.


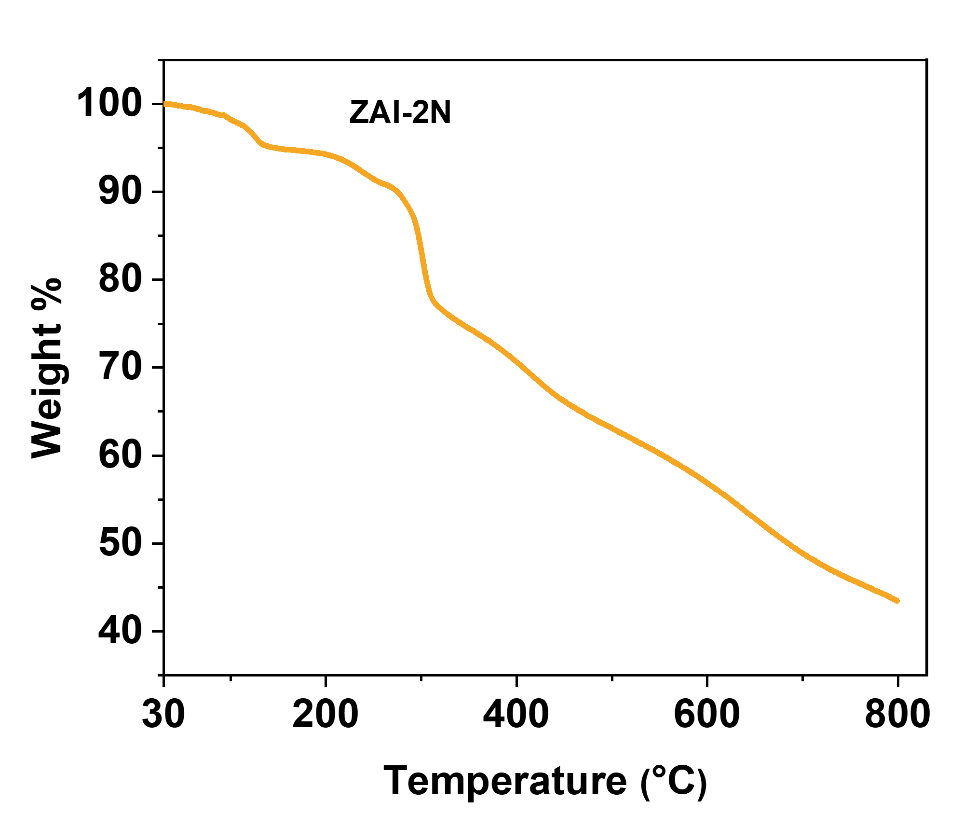


**Figure S14** TGA curve for **ZAI-2N**.

**Figure S15** TGA curve for **ZAI-3N**.

**Figure S16** (a) N_2_ adsorption-desorption isotherms at 77 K for **ZAI**; (b) NLDFT pore size distributions of **ZAI**.


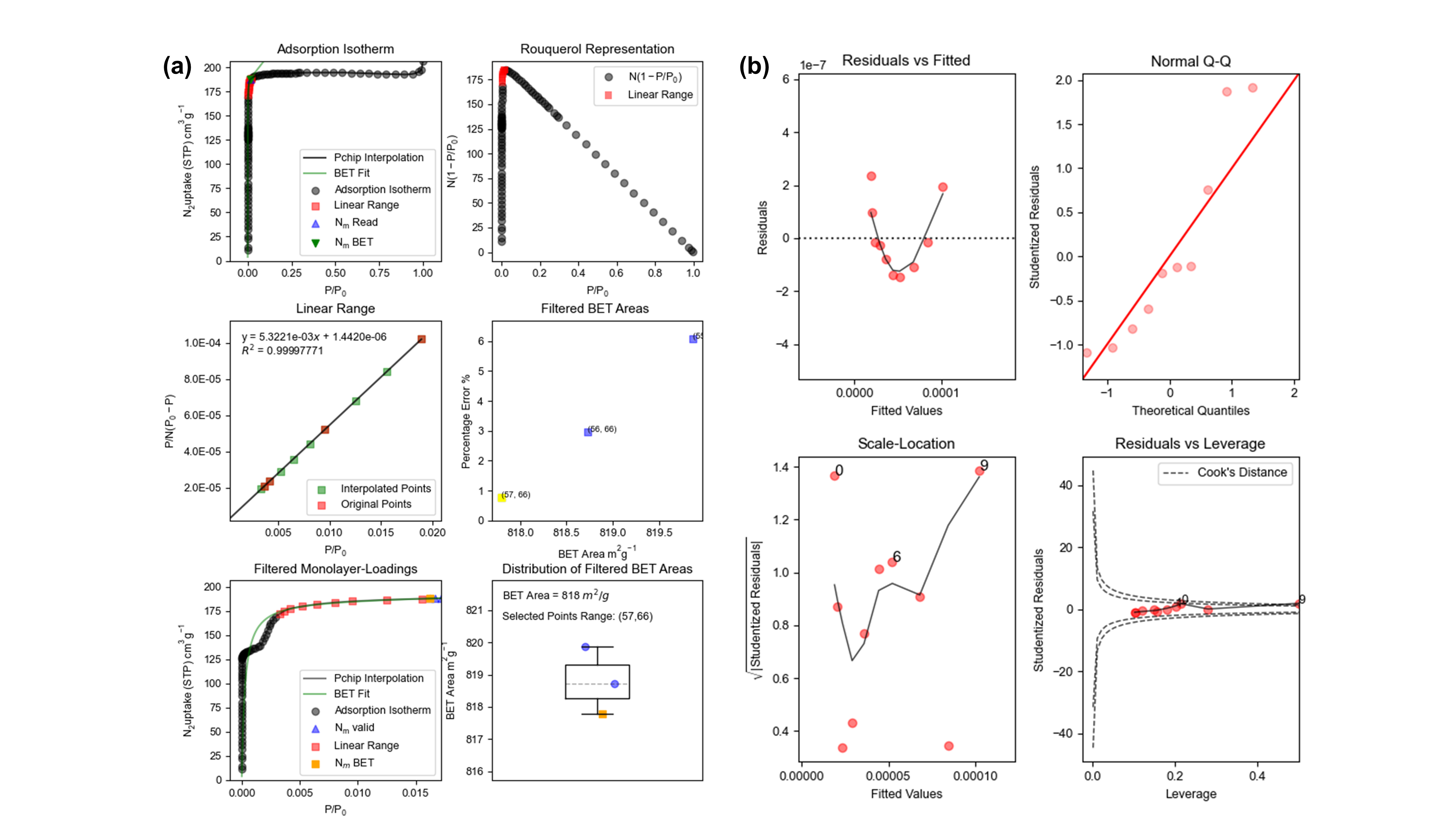


**Figure S17** (a) BETSI analyses and (b) regression diagnostics for **ZAI** by using 77 K N_2_ adsorption isotherm.

**Figure S18** (a) N_2_ adsorption-desorption isotherms at 77 K for **ZAI-3M**; (b) CO_2_ adsorption-desorption isotherms at 195 K for **ZAI-3M.**


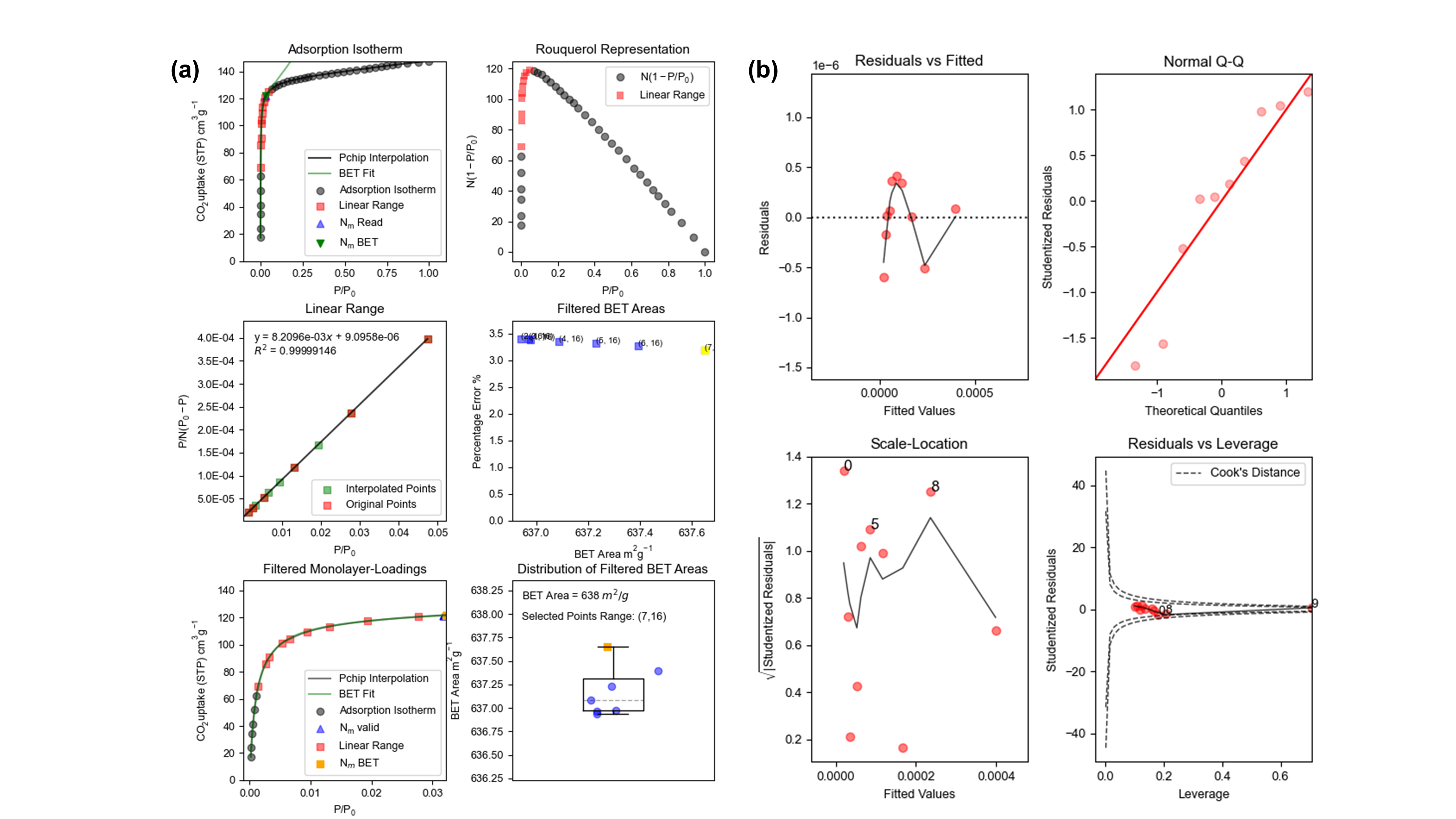


**Figure S19** (a) BETSI analyses and (b) regression diagnostics for **ZAI-3M** by using 195 K CO_2_ adsorption isotherm.

**Figure S20** Pore size distribution of **ZAI-3M** calculated based on CO_2_ adsorption isotherms at 195 K.

**Figure S21** (a) N_2_ adsorption-desorption isotherms at 77 K for **ZAI-2N**; (b) CO_2_ adsorption-desorption isotherms at 195 K for **ZAI-2N.**


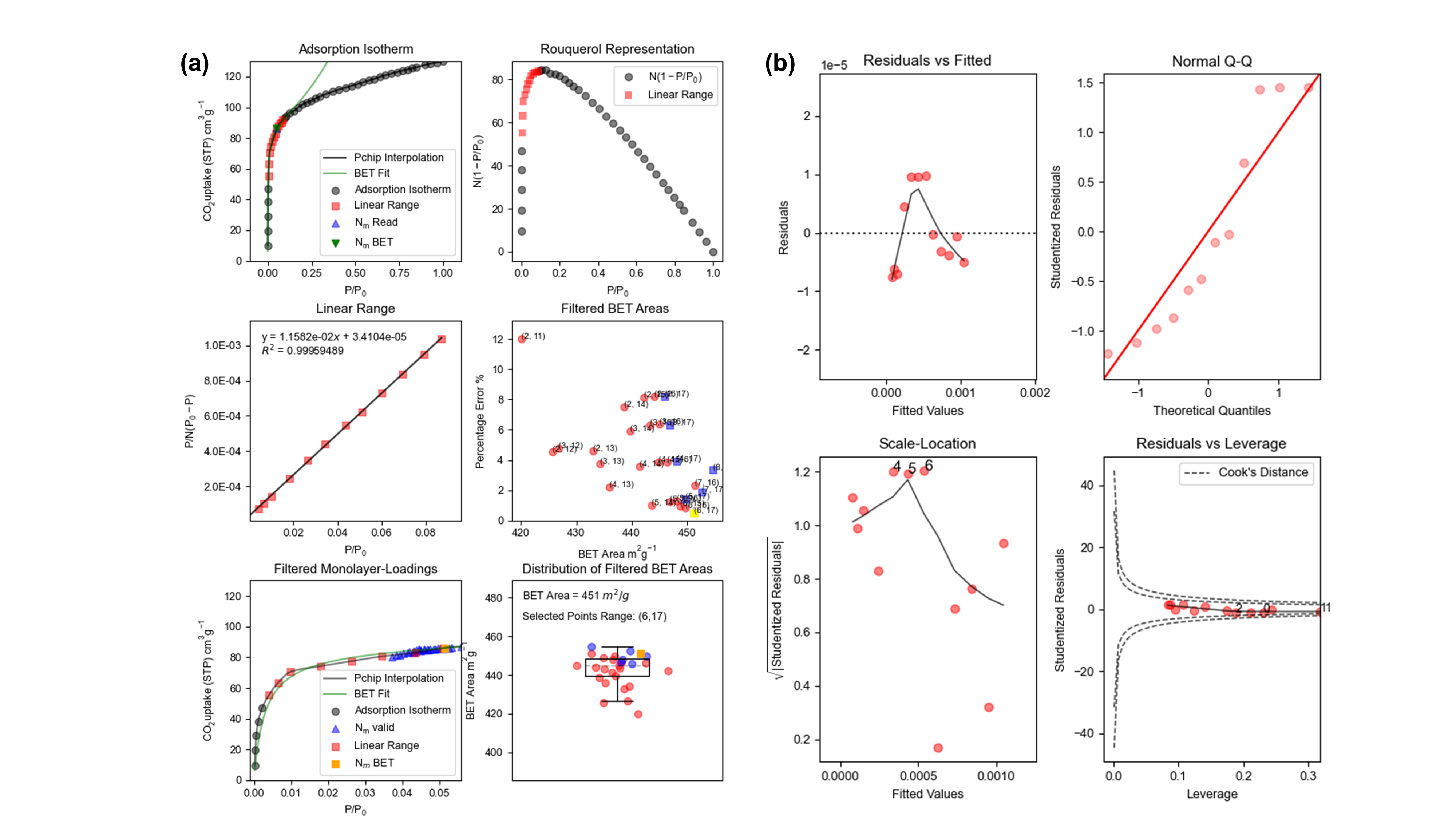


**Figure S22** (a) BETSI analyses and (b) regression diagnostics for **ZAI-2N** by using 195 K CO_2_ adsorption isotherm.

**Figure S23** Pore size distribution of **ZAI-2N** calculated based on CO_2_ adsorption isotherms at 195 K.

**Figure S24** (a) N_2_ adsorption-desorption isotherms at 77 K for **ZAI-3N**; (b) CO_2_ adsorption-desorption isotherms at 195 K for **ZAI-3N.**


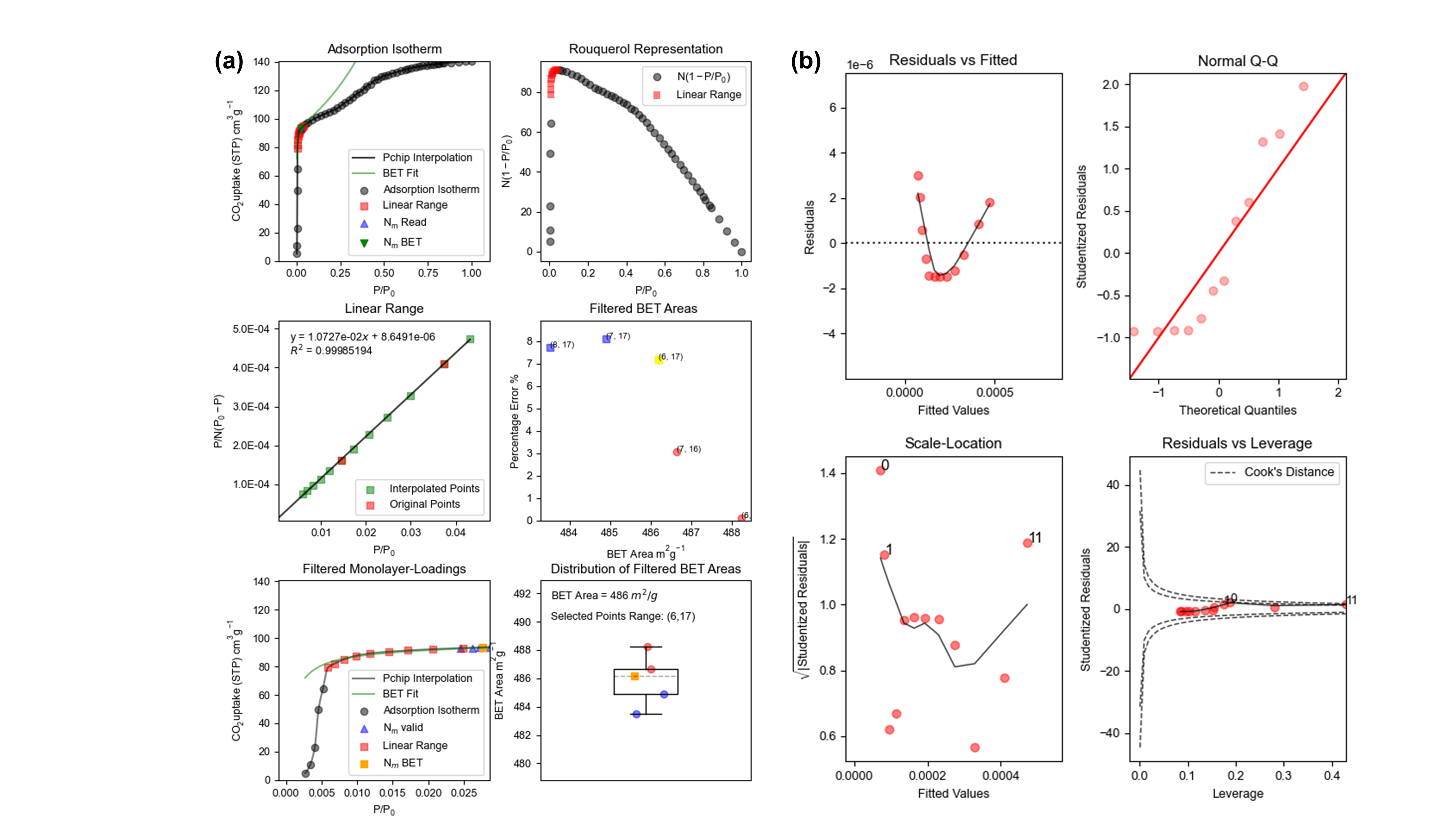


**Figure S25** (a) BETSI analyses and (b) regression diagnostics for **ZAI-3N** by using 195 K CO_2_ adsorption isotherm.

**Figure S26** Pore size distribution of **ZAI-3N** calculated based on CO_2_ adsorption isotherms at 195 K.

**Figure S27** Cycling adsorption and adsorption performance of **ZAI-3N** for C_2_H_6_ at 313 K and 100 kPa.

**Figure S28** Single-component C_2_H_6_ and C_2_H_4_ adsorption isotherms of **ZAI** at (a) 298 K and (b) 273 K and 100 kPa.

**Figure S29** Single-component C_2_H_6_ and C_2_H_4_ adsorption isotherms of **ZAI-3M** at (a) 273 K. (b) 298 K, (c) 313 K and 100 kPa.

**Figure S30** Single-component C_2_H_6_ and C_2_H_4_ adsorption isotherms of **ZAI-2N** at (a) 298K, (b) 273 K and 100 kPa.

**Figure S31** The IAST selectivity data calculated with C_2_H_6_/C_2_H_4_ ratio of 1/9 for **ZAI** at 298 K.

**Figure S32** The IAST selectivity data calculated with C_2_H_6_/C_2_H_4_ ratio of 1/9 for **ZAI-3M** at 298 K.

**Figure S33** The IAST selectivity data calculated with C_2_H_6_/C_2_H_4_ ratio of 1/9 for **ZAI-2N** at 298 K.

.

**Figure S34** The IAST selectivity data calculated with C_2_H_6_/C_2_H_4_ ratio of 1/9 for **ZAI-3N** at 298 K.


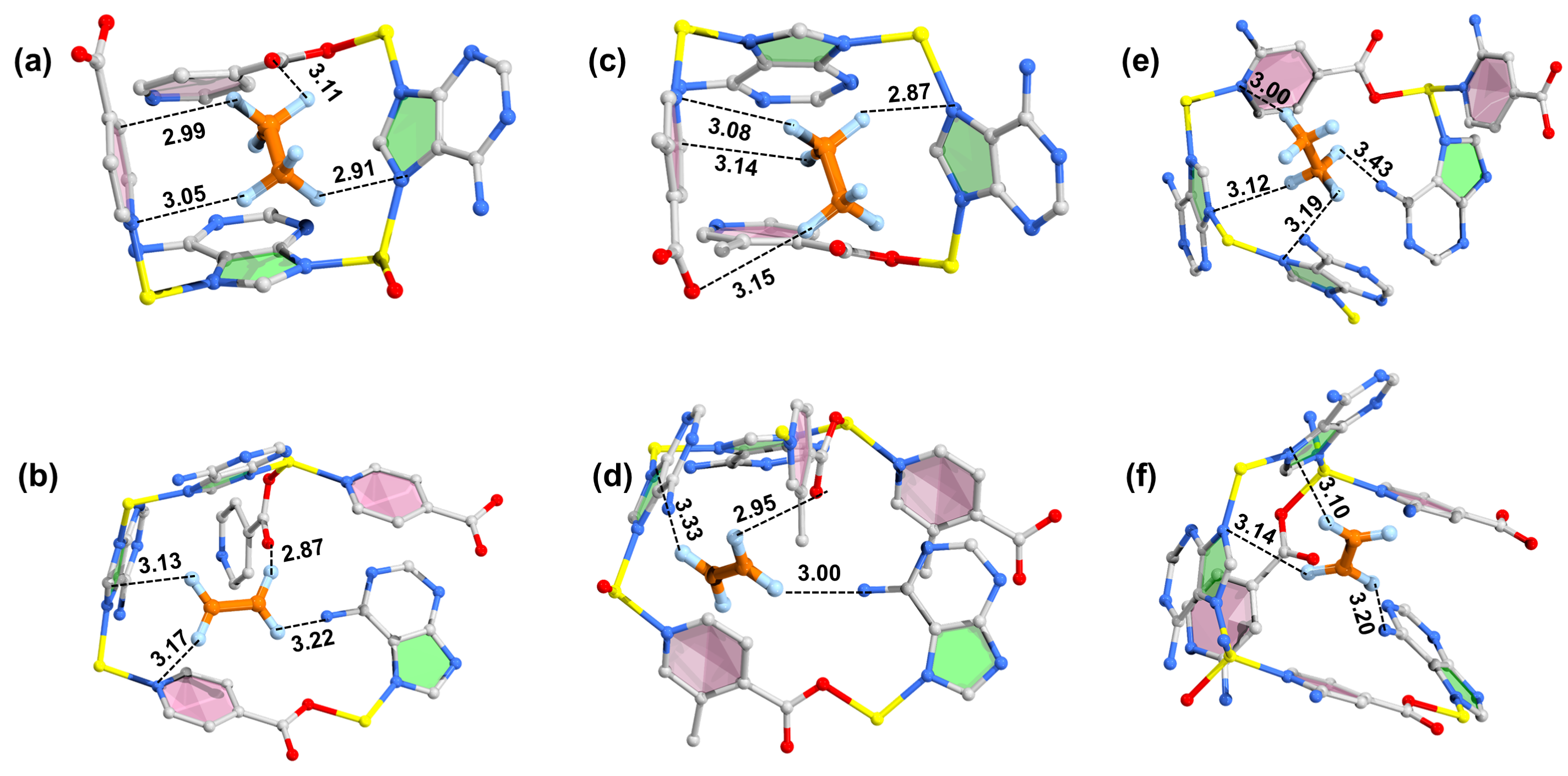


**Figure S35** Calculated binding sites of (a) C_2_H_6_, (b) C_2_H_4_ in **ZAI**; (c) C_2_H_6_, (d) C_2_H_4_ in **ZAI-3M**; (e) C_2_H_6_, (f) C_2_H_4_ in **ZAI-2N**.

**Figure S36** Energy profile of C₂H_4_ diffusion pathway in **ZAI-3N**.

**
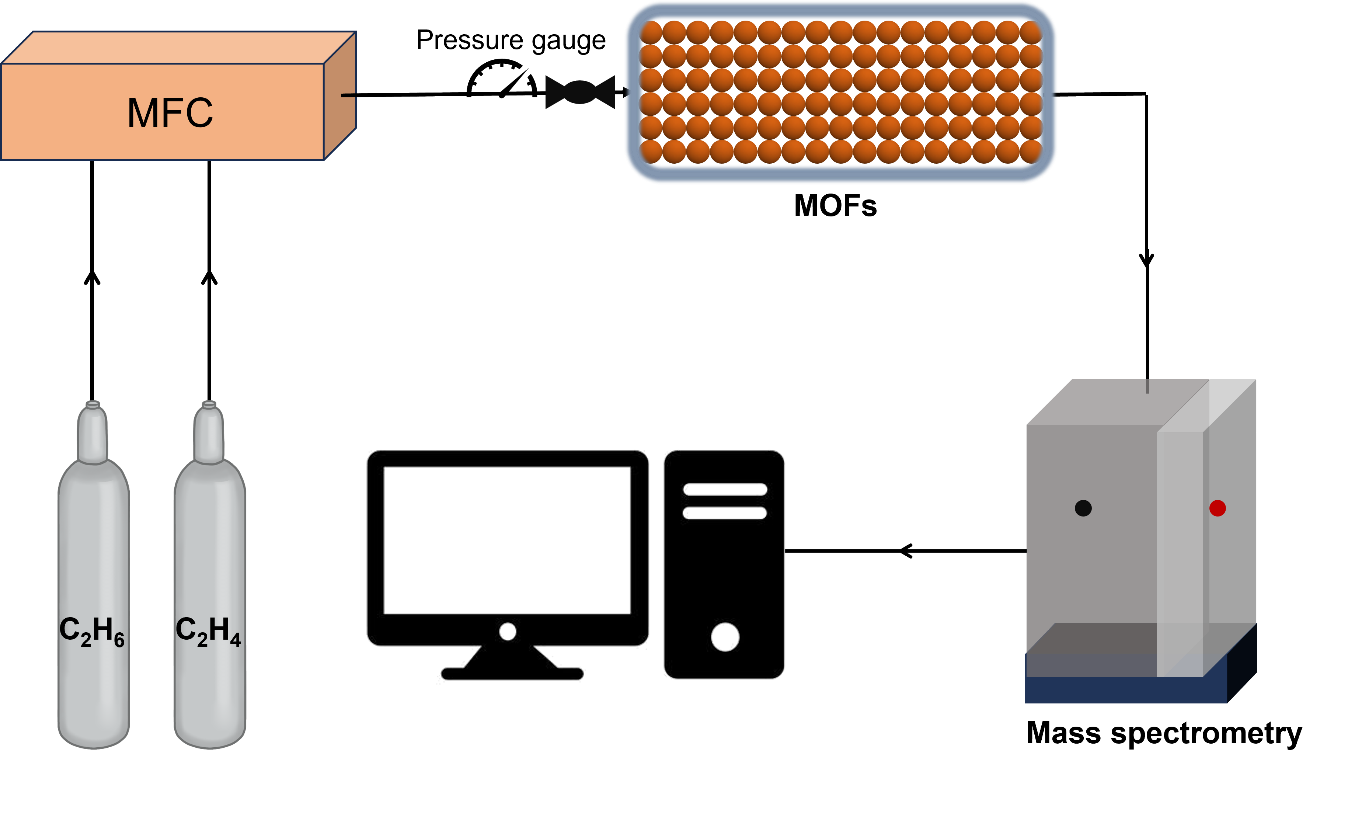
**

**Figure S37** The breakthrough experimental set-up schematic.

**Figure S38** Experimental breakthrough curves for C_2_H_6_/C_2_H_4_ (6 mL min^-1^, 5/5, v/v) mixture separation through a packed bed of ZAI-3N at 313 K and 1 bar.

**Figure S39** Water vapor adsorption and desorption isotherms of ZAI-3N measured at 298 K.

**Figure S40** Dual-site Langmuir-Freundlich model for (a) C_2_H_6_ and (b) C_2_H_4_ adsorption isotherm on **ZAI** at 298 K.

**Figure S41** Dual-site Langmuir-Freundlich model for (a) C_2_H_6_ and (b) C_2_H_4_ adsorption isotherm on **ZAI-3M** at 298 K.

**Figure S42** Dual-site Langmuir-Freundlich model for (a) C_2_H_6_ and (b) C_2_H_4_ adsorption isotherm on **ZAI-2N** at 298 K.

**Figure S43** Dual-site Langmuir-Freundlich model for (a) C_2_H_6_ and (b) C_2_H_4_ adsorption isotherm on **ZAI-3N** at 298 K.

**Figure S44** Dual-site Langmuir-Freundlich model for (a) C_2_H_6_ and (b) C_2_H_4_ adsorption isotherm on **ZAI-3N** at 313 K.

**Table S2 Crystallographic data**

| **Compound** | **ZAI-3M** | **ZAI-3N** |
| --- | --- | --- |
| CCDC | 2476335 | 2476056 |
| Formula | C_15_H_17_N_7_O_3_Zn | C_14_H_16_N_8_O_3_Zn |
| *D_calc._*/ g cm^-3^ | 1.528 | 1.533 |
| μ/mm^‑1^ | 1.413 | 1.416 |
| Formula Weight | 408.72 | 409.72 |
| *T*/K | 150.00(10) | 150.00(10) |
| Crystal System | Orthorhombic | Orthorhombic |
| Flack Parameter | -0.03(2) | 0.035(17) |
| Space Group | *Pna2_1_* | *Pna*2_1_ |
| *a*/Å | 17.525(2) | 17.2368(6) |
| *b*/Å | 11.2494(14) | 11.6753(5) |
| *c*/Å | 9.0136(12) | 8.8219(4) |
| *α*/° | 90 | 90 |
| *β*/° | 90 | 90 |
| *γ*/° | 90 | 90 |
| *V*/Å^3^ | 1777.0(4) | 1775.36(13) |
| *Z* | 4 | 4 |
| GoF | 1.047 | 1.041 |
| *R_1_* (>2σ) | 0.0575 | 0.0332 |
| *wR_2_ (>2*σ*)* | 0.1431 | 0.0780 |

**Table S3. Comparison of C_2_H_6_ and C_2_H_4_ uptake ration among representative MOFs at 298 K and 1 bar.**

| Compounds | Uptake (mmol/g) | | Ratio of uptake | IAST  Selectivity (5/5) | Conditions | Refs |
| --- | --- | --- | --- | --- | --- | --- |
|  | C_2_H_6_ | C_2_H_4_ | C_2_H_6_/C_2_H_4_ |  |  |  |
| **ZAI-3N** | 2.46 | 0.23 | 10.60 | 11.70 | 313 K | This work |
| **ZAI-3N** | 2.39 | 0.92 | 2.58 | 4.64 | 298 K | This work |
| **Cu(Qc)_2_** | 1.85 | 0.78 | 2.37 | 3.4 |  | ^[16]^ |
| **NKMOF-14-PZ** | 5.64 | 3.44 | 1.64 | 1.89 |  | ^[17]^ |
| **NKMOF-14-PD** | 5.35 | 3.39 | 1.59 | 1.96 |  | ^[17]^ |
| **NPU-3** | 3.33 | 2.19 | 1.52 | 3.21 |  | ^[18]^ |
| **Ni(bdc)(ted)_0.5_** | 5.0 | 3.4 | 1.47 | 1.7 |  | ^[19]^ |
| **Mn-PNMI** | 2.75 | 2.02 | 1.36 | 1.38 |  | ^[20]^ |
| **ZIF-8** | 2.52 | 1.414 | 1.33 | 1.70 |  | ^[21]^ |
| **MIL-142A** | 3.8 | 2.9 | 1.31 |  |  | ^[22]^ |
| **Fe_2_(O_2_)(dobdc)** | 3.32 | 2.55 | 1.3 | 4.4 |  | ^[23]^ |
| **ZJU-120a** | 4.91 | 3.93 | 1.25 | 2.74 | 296 K | ^[24]^ |
| **(Hf)DUT-52a** | 4.02 | 3.22 | 1.25 | 1.9 |  | ^[25]^ |
| **PCN-250** | 5.21 | 4.22 | 1.23 | 1.9 |  | ^[26]^ |
| **NKMOF-8-Br** | 4.22 | 3.67 | 1.15 | 2.65 |  | ^[27]^ |
| **JNU-2** | 4.1 | 3.6 | 1.14 | 1.6 |  | ^[28]^ |
| **MUF-15** | 4.69 | 4.15 | 1.13 | 1.96 | 296 K | ^[29]^ |
| **MCIF-1** | 2.40 | 2.19 | 1.10 | 1.61 |  | ^[30]^ |
| **Fe_2_(BDP)_3_** | 2.25 | 2.11 | 1.07 | 2.1 |  | ^[31]^ |
| **NTU-25** | 4.38 | 4.24 | 1.03 |  |  | ^[32]^ |
| **MAF-49** | 1.72 | 1.69 | 1.02 | 2.71 |  | ^[33]^ |
| **ZIF-7** | 1.83 | 1.80 | 1.02 | 1.46 |  | ^[34]^ |
| **ZU-925** | 2.82 | 2.40 | 1.18 | 2.82 |  | ^[35]^ |
| **PCP-IPA** | 2.50 | 2.13 | 1.17 | 2.80 |  | ^[36]^ |
| **ZAI** | 2.68 | 2.65 | 1.01 | 1.83 |  | This work |
| **ZAI-3M** | 2.82 | 2.83 | 1.00 | 2.37 |  | This work |
| **ZAI-2N** | 2.61 | 2.75 | 0.95 | 1.43 |  | This work |

**References**

[1] F. Wang, Y.-X. Tan, H. Yang, H.-X. Zhang, Y. Kang, J. Zhang, "A new approach towards tetrahedral imidazolate frameworks for high and selective CO 2 uptake," *Chem. Commun.* **2011**, *47*, 5828-5830.

[2] G. M. Sheldrick, "Crystal structure refinement with SHELXL," *Crystal Structure Communications* **2015**, *71*, 3-8.

[3] G. M. Sheldrick, "A short history of SHELX," *Foundations of crystallography* **2008**, *64*, 112-122.

[4] A. A. Coelho, "TOPAS and TOPAS-Academic: an optimization program integrating computer algebra and crystallographic objects written in C++," *Applied Crystallography* **2018**, *51*, 210-218.

[5] Kresse G, Furthmüller J. Efficient iterative schemes for ab initio total-energy calculations using a plane-wave basis set[J]. *Phys. Rev. B*, **1996**, *54*, 11169.

[6] J. P. Perdew, K. Burke, M. Ernzerhof, "Generalized gradient approximation made simple," *Phys. Rev. Lett.* **1996**, *77*, 3865.

[7] A. D. Becke, E. R. Johnson, "A density-functional model of the dispersion interaction," *The Journal of chemical physics* **2005**, *123*.

[8] S. Grimme, S. Ehrlich, L. Goerigk, "Effect of the damping function in dispersion corrected density functional theory," *J. Comput. Chem.* **2011**, *32*, 1456-1465.

[9] Grimme S, Antony J, Ehrlich S, et al. A consistent and accurate ab initio parametrization of density functional dispersion correction (DFT-D) for the 94 elements H-Pu[J]. *J. Chem. Phys.* **2010**, *132*, 154104.

[10] G. Henkelman, B. P. Uberuaga, H. Jónsson, "A climbing image nudged elastic band method for finding saddle points and minimum energy paths," *J. Chem. Phys.* **2000**, *113*, 9901-9904.

[11] G. Mills, H. Jónsson, G. K. Schenter, "Reversible work transition state theory: application to dissociative adsorption of hydrogen," *Surf. Sci.* **1995**, *324*, 305-337.

[12] J. W. Osterrieth, J. Rampersad, D. Madden, N. Rampal, L. Skoric, B. Connolly, M. D. Allendorf, V. Stavila, J. L. Snider, R. Ameloot, "How reproducible are surface areas calculated from the BET equation?," *Adv. Mater.* **2022**, *34*, 2201502.

[13] A. Rappe, K. Colwell, C. Casewit, "Application of a universal force field to metal complexes," *Inorg. Chem.* **1993**, *32*, 3438-3450.

[14] N. Metropolis, A. W. Rosenbluth, M. N. Rosenbluth, A. H. Teller, E. Teller, "Equation of state calculations by fast computing machines," *The journal of chemical physics* **1953**, *21*, 1087-1092.

[15] B. Delley, "From molecules to solids with the DMol 3 approach," *The Journal of chemical physics* **2000**, *113*, 7756-7764.

[16] R.-B. Lin, H. Wu, L. Li, X.-L. Tang, Z. Li, J. Gao, H. Cui, W. Zhou, B. Chen, "Boosting ethane/ethylene separation within isoreticular ultramicroporous metal–organic frameworks," *J. Am. Chem. Soc.* **2018**, *140*, 12940-12946.

[17] W. Liu, S. Geng, N. Li, S. Wang, S. Jia, F. Jin, T. Wang, K. A. Forrest, T. Pham, P. Cheng, "Highly Robust Microporous Metal‐Organic Frameworks for Efficient Ethylene Purification under Dry and Humid Conditions," *Angew. Chem. Int. Ed.* **2022**.

[18] B. Zhu, J.-W. Cao, S. Mukherjee, T. Pham, T. Zhang, T. Wang, X. Jiang, K. A. Forrest, M. J. Zaworotko, K.-J. Chen, "Pore engineering for one-step ethylene purification from a three-component hydrocarbon mixture," *J. Am. Chem. Soc.* **2021**, *143*, 1485-1492.

[19] W. Liang, F. Xu, X. Zhou, J. Xiao, Q. Xia, Y. Li, Z. Li, "Ethane selective adsorbent Ni (bdc)(ted) 0.5 with high uptake and its significance in adsorption separation of ethane and ethylene," *Chem. Eng. Sci.* **2016**, *148*, 275-281.

[20] L. Yang, Y. Wang, Y. Chen, J. Yang, X. Wang, L. Li, J. Li, "Microporous metal-organic framework with specific functional sites for efficient removal of ethane from ethane/ethylene mixtures," *Chem. Eng. J.* **2020**, *387*, 124137.

[21] H. Bux, C. Chmelik, R. Krishna, J. Caro, "Ethene/ethane separation by the MOF membrane ZIF-8: molecular correlation of permeation, adsorption, diffusion," *J. Membr. Sci.* **2011**, *369*, 284-289.

[22] Y. Chen, H. Wu, D. Lv, R. Shi, Y. Chen, Q. Xia, Z. Li, "Highly adsorptive separation of ethane/ethylene by an ethane-selective MOF MIL-142A," *Industrial & Engineering Chemistry Research* **2018**, *57*, 4063-4069.

[23] L. Li, R.-B. Lin, R. Krishna, H. Li, S. Xiang, H. Wu, J. Li, W. Zhou, B. Chen, "Ethane/ethylene separation in a metal-organic framework with iron-peroxo sites," *Science* **2018**, *362*, 443-446.

[24] J. Pei, J.-X. Wang, K. Shao, Y. Yang, Y. Cui, H. Wu, W. Zhou, B. Li, G. Qian, "Engineering microporous ethane-trapping metal–organic frameworks for boosting ethane/ethylene separation," *J. Mater. Chem. A* **2020**, *8*, 3613-3620.

[25] X.-W. Gu, J. Pei, K. Shao, H.-M. Wen, B. Li, G. Qian, "Chemically stable hafnium-based metal–organic framework for highly efficient C2H6/C2H4 separation under humid conditions," *ACS Appl. Mater. Interfaces* **2021**, *13*, 18792-18799.

[26] Y. Chen, Z. Qiao, H. Wu, D. Lv, R. Shi, Q. Xia, J. Zhou, Z. Li, "An ethane-trapping MOF PCN-250 for highly selective adsorption of ethane over ethylene," *Chem. Eng. Sci.* **2018**, *175*, 110-117.

[27] S. Geng, E. Lin, X. Li, W. Liu, T. Wang, Z. Wang, D. Sensharma, S. Darwish, Y. H. Andaloussi, T. Pham, "Scalable Room-Temperature Synthesis of Highly Robust Ethane-Selective Metal–Organic Frameworks for Efficient Ethylene Purification," *J. Am. Chem. Soc.* **2021**.

[28] H. Zeng, X.-J. Xie, M. Xie, Y.-L. Huang, D. Luo, T. Wang, Y. Zhao, W. Lu, D. Li, "Cage-Interconnected Metal–Organic Framework with Tailored Apertures for Efficient C2H6/C2H4 Separation under Humid Conditions," *J. Am. Chem. Soc.* **2019**, *141*, 20390-20396.

[29] O. T. Qazvini, R. Babarao, Z.-L. Shi, Y.-B. Zhang, S. G. Telfer, "A robust ethane-trapping metal–organic framework with a high capacity for ethylene purification," *J. Am. Chem. Soc.* **2019**, *141*, 5014-5020.

[30] N. Zhao, P. Li, X. Mu, C. Liu, F. Sun, G. Zhu, "Facile synthesis of an ultra-stable metal–organic framework with excellent acid and base resistance," *Faraday Discuss.* **2017**, *201*, 63-70.

[31] Q. Gao, A.-L. Li, X. Chen, N. Lu, Y.-M. Zhang, L.-Z. Chen, "A microporous metal–organic framework with triangular channels for C2H6/C2H4 adsorption separation," *Sep. Purif. Technol.* **2021**, *276*, 119424.

[32] H. Cao, Z. Lu, K. Hyeon-Deuk, I.-Y. Chang, Y. Wang, Z. Xin, J. Duan, W. Jin, "Enhanced breakthrough efficiency by a chemically stable porous coordination polymer with optimized nanochannel," *ACS Appl. Mater. Interfaces* **2018**, *10*, 39025-39031.

[33] P.-Q. Liao, W.-X. Zhang, J.-P. Zhang, X.-M. Chen, "Efficient purification of ethene by an ethane-trapping metal-organic framework," *Nat. Commun.* **2015**, *6*, 1-9.

[34] C. Gucuyener, J. Van Den Bergh, J. Gascon, F. Kapteijn, "Ethane/ethene separation turned on its head: selective ethane adsorption on the metal− organic Framework ZIF-7 through a gate-opening mechanism," *J. Am. Chem. Soc.* **2010**, *132*, 17704-17706.

[35] P. Zhang, D. Zhou, X. Suo, X. Cui, L. Yang, H. Xing, "Tailored Synergistic Binding Environment in Metal‐Organic Frameworks for Record One‐Step Ethylene Purification from Multicomponent Mixtures," *Angew. Chem.* **2025**, *137*, e18996.

[36] P. Zhang, L. Yang, X. Liu, J. Wang, X. Suo, L. Chen, X. Cui, H. Xing, "Ultramicroporous material based parallel and extended paraffin nano-trap for benchmark olefin purification," *Nat. Commun.* **2022**, *13*, 4928.
